# *De novo* transcriptome assembly and analysis of differential gene expression in response to drought in European beech

**DOI:** 10.1371/journal.pone.0184167

**Published:** 2017-09-05

**Authors:** Markus Müller, Sarah Seifert, Torben Lübbe, Christoph Leuschner, Reiner Finkeldey

**Affiliations:** 1 Forest Genetics and Forest Tree Breeding, Faculty for Forest Sciences and Forest Ecology, University of Goettingen, Goettingen, Lower-Saxony, Germany; 2 Plant Ecology and Ecosystems Research, Albrecht von Haller Institute for Plant Sciences, University of Goettingen, Goettingen, Lower-Saxony, Germany; 3 University of Kassel, Kassel, Hesse, Germany; University of Western Sydney, AUSTRALIA

## Abstract

Despite the ecological and economic importance of European beech (*Fagus sylvatica* L.) genomic resources of this species are still limited. This hampers an understanding of the molecular basis of adaptation to stress. Since beech will most likely be threatened by the consequences of climate change, an understanding of adaptive processes to climate change-related drought stress is of major importance. Here, we used RNA-seq to provide the first drought stress-related transcriptome of beech. In a drought stress trial with beech saplings, 50 samples were taken for RNA extraction at five points in time during a soil desiccation experiment. *De novo* transcriptome assembly and analysis of differential gene expression revealed 44,335 contigs, and 662 differentially expressed genes between the stress and normally watered control group. Gene expression was specific to the different time points, and only five genes were significantly differentially expressed between the stress and control group on all five sampling days. GO term enrichment showed that mostly genes involved in lipid- and homeostasis-related processes were upregulated, whereas genes involved in oxidative stress response were downregulated in the stressed seedlings. This study gives first insights into the genomic drought stress response of European beech, and provides new genetic resources for adaptation research in this species.

## Introduction

The climate of Central Europe has significantly warmed during the past 40 years and is expected to continue to do so in the decades to come [1]. Severe and recurrent droughts have been identified as a major threat to the vitality and productivity of European forests, including Central European beech forests, which mainly occur in a humid sub-oceanic climate [2, 3]. European beech (*Fagus sylvatica* L.) is considered to be more drought sensitive than most other deciduous tree species of the region [4–7]. Thus, this species may face growth reductions and perhaps increased mortality under a warmer and summer-drier climate in various regions of Central Europe [8, 9], and the investigation of its adaptation potential to changing environmental conditions and the mechanism of drought tolerance are of great importance. Several studies revealed physiological and morphological differences among beech populations of different origin for drought related traits [10–17]. This suggests that beech trees may differ with respect to drought adaptation, which offers a potential for selecting genotypes with better performance under climate warming. Nevertheless, knowledge about the molecular basis of drought stress tolerance is still scarce for this species. Existing studies about the genetic basis of drought stress tolerance in beech studied on only a few potential drought stress genes. Despite its relatively small genome size (ca. 540 Mbp; [18]) and its economic and ecological importance, genomic resources of beech are in fact still very limited [19]. The *F*. *sylvatica* genome has not been sequenced yet, and to date, there is only one transcriptome (related to dormancy regulation; [19]) available for this species. Therefore, more genomic resources are needed to understand the molecular basis of adaptation.

Here, we report the first drought stress-related transcriptome of beech. A drought stress experiment with saplings under controlled conditions was conducted, and samples from five stressed plants and five well-watered control plants were taken at five different points in time over the course of the experiment for sequencing. This study gives first insights into the genomic drought stress response of beech. Additionally, new genomic resources for beech including new candidate genes for drought stress tolerance are reported, which can be used in further studies.

## Materials and methods

### Drought stress experiment, sample collection, and genotyping with microsatellite markers

The beech plants used in this study were part of a larger drought stress experiment as described in [20]. Briefly, 1- to 2-year old saplings were obtained from a nursery close to Göttingen (Germany), and cultivated under uniform conditions for 16 months. Five saplings each were grown in pots with a diameter of 0.58 m (0.05 m^3^ volume) with equal distances among plants. The pots were placed outdoors under a rain shelter made of transparent plexiglass allowing to control water supply, while the microclimate was close to natural conditions. The experiment consisted of a moist and a dry treatment. Drought was applied in the period July to September 2011, and May to August 2012. By regular irrigation (every 3 to 5 days), the volumetric soil water content (SWC) of the pots was kept more or less constant during the experiment, i.e. fluctuated moderately below target values of maximal SWC of ca. 21% (95% of field capacity in the soil) in the moist treatment, and ca. 12% (57% of field capacity) in the drought treatment. In total, 10 plants (5 plants of the drought stress group, and 5 plants of the control group) were used for the present study. Stomatal conductance (*G*_*S*_, mmol m^-2^ s^-1^) was measured in 2012 on June 28, July 5, July 12, July 19, and July 26 around noon (ca. 11 a.m. to 2 p.m.) to infer the intensity of drought stress. The measurements were conducted with a porometer (Delta-T Devices, Cambridge, UK) on each two fully developed leaves per plant. The leaves were tagged and repeatedly measured on the five selected sampling days in the year 2012 (June 28, July 5, July 12, July 19, and July 26), always on the last day before the next irrigation event. Statistically significant differences between the drought and control group were identified with the non-parametric Mann-Whitney U-test implemented in STATISTICA 12.5 (StatSoft, Tulsa, USA). Two leaves per tree were sampled at every sampling day, immediately frozen in liquid nitrogen, and stored at -60°C until RNA extraction.

Since the saplings of the drought stress experiment were obtained from a nursery, SSR genotyping was used to infer the neutral genetic structure among the ten selected individuals for RNA-seq. Total DNA was extracted from leaves not used for RNA extraction with a DNeasy 96 Plant Kit (QIAGEN, Hilden, Germany). The amount and quality of DNA were analyzed by 1% agarose gel electrophoreses with 1 X TAE as running buffer. SSR genotyping was conducted as described in [21]. Briefly, nine highly polymorphic SSR markers including three EST microsatellite markers were used [22–25]. Primers were labeled with fluorescent dyes and pooled into three different sets for multiplexing. After PCR, the SSR fragments were separated on an ABI 3130xl Genetic Analyzer (Applied Biosystems, Foster City, USA), and scored using GeneMapper 4.0 (Applied Biosystems, Foster City, USA). Genetic diversity indices observed heterozygosity (H_o_), expected heterozygosity (H_e_), and fixation index (F) were calculated with GenAlEx 6.5 [26, 27]. Statistic differences between the drought and control group for the genetic diversity indices were tested using the Mann-Whitney U-test implemented in STATISTICA 12.5 (StatSoft, Tulsa, USA).

### Sample preparation and RNA sequencing

In total, 50 samples (10 plants on five sampling days) were used for RNA extraction. Total RNA was extracted using the RNeasy Plant Mini Kit (QIAGEN, Hilden, Germany). Extracted RNA was sent to Chronix Biomedical GmbH (Göttingen, Germany) for library preparation and sequencing. RNA quality and integrity were evaluated using an Agilent 2100 Bioanalyzer (Agilent Technologies, Santa Clara, CA, USA). cDNA library preparations were conducted using the NEBNext Ultra RNA Library Prep Kit for Illumina (New England BioLabs, Frankfurt am Main, Germany). Additionally, a normalized composite sample was created to enhance the *de novo* transcriptome assembly. For that, extracted RNA of all 50 samples was pooled to a single composite sample. The cDNA library was prepared using the Mint-2 cDNA synthesis kit (Evrogen, Moscow, Russia), and normalized using the Trimmer-2 cDNA normalization kit (Evrogen, Moscow, Russia). The 51 libraries (50 single samples from the five time points plus one composite sample) were paired-end sequenced on five lanes on an Illumina HiSeq2000 platform (Illumina, San Diego, CA, USA). Each library was uniquely tagged with a barcode to allow library pooling for sequencing, and control and treatment samples were always sequenced together on one lane.

### *De novo* transcriptome assembly, read mapping and sequence annotation

The *de novo assembly* was conducted with the CLC Genomics workbench 7.04 (CLC bio, Aarhus, Denmark) based on a *de Bruijn graphs* approach. Sequencing adapters were removed and the sequence reads were further trimmed for quality and ambiguity. The *de novo* assembly was conducted based on the composite sample using default parameters in the CLC Genomics workbench. The program cd-hit-est [28] with a sequence identity threshold of 0.95 was used to reduce the redundancy of the assembly. Finally, reads of the 50 samples of the different sampling days were mapped to the newly created assembly. Sequence annotation was carried out with the software BLAST2GO [29]. For that, contigs were searched against the NCBI non-redundant (nr) protein database using BLASTX [30] with an E-value cut-off of 1e^-3^. Based on these results, gene ontology (GO) terms [31] were assigned to the sequences. GO-slim mapping against the plant slim file (The Arabidopsis Information Resource (TAIR), http://www.arabidopsis.org) using BLAST2GO [32] was conducted to give an overview of the GO term distribution over the entire transcriptome.

### Identification of differential gene expression

For the identification of differentially expressed genes (DEGs) between the stress and control group, two different methods were used: edgeR 3.4.0 [33] implemented in the CLC Genomics Workbench, and the R/Bioconductor package DESeq2 1.12.4. [34]. For both methods, genes with a FDR (false discovery rate) <0.1 [35] were considered to be differentially expressed. Analyzes were carried out for each of the five time points separately. A GO term enrichment analysis was performed to identify functional categories enriched in DEGs between the stress and control group. For that, the software BLAST2GO [29] with Fisher’s exact test was used. A FDR [35] threshold of 0.05 was applied.

### Quantitative real-time PCR

For the confirmation of differential gene expression revealed by RNA-seq, quantitative real-time PCR was used. In total, 12 samples (six stressed plants and six control plants) from the fifth sampling day were used for qPCR validation. The samples included eight plants, which were also used for RNA-seq and four additional plants of the drought stress experiment (two plants of the stress group and two plants of the control group), which were not included in the RNA-seq analysis. Total RNA was extracted as described for the RNA-seq experiment, and 500 ng RNA was used for cDNA synthesis using the SuperScript III First-Strand Synthesis System for RT-PCR (Invitrogen, Carlsbad, CA, USA) using Oligo(dT)_20_ primer. Genes for validation were selected based on their fold chance and biological function. Gene specific primers were designed using Primer-BLAST [36] (S1 File). *Actin* was used as a reference gene and primers were obtained from [37]. Identity of the target sequences was confirmed by sequencing of PCR-products. RT-PCR was performed in a TOptical 96 Thermocycler (Biometra, Göttingen, Germany) with three technical replicates for each sample. Each well included 4 μL HPCL-grade H_2_O, 10 μL innuMIX qPCR MasterMix SyGreen (Analytik Jena, Jena, Germany), 2.5 μL of forward and reverse primers (5 pmol), and 1 μL diluted cDNA (1:10). The PCR program comprised the following steps: pre-incubation for 3 min at 95°C, 45 cycles of amplification for 5 s at 95°C, 5 s at 58°C, and 15 s at 72°C. Relative gene expression was calculated with the software GenEx 6.1 (MultiD Analyses AB, Göteborg, Sweden). Primer efficiencies were determined by dilution series for the analyzed genes.

## Results

### Drought stress experiment and SSR genotyping

**Fig 1:**
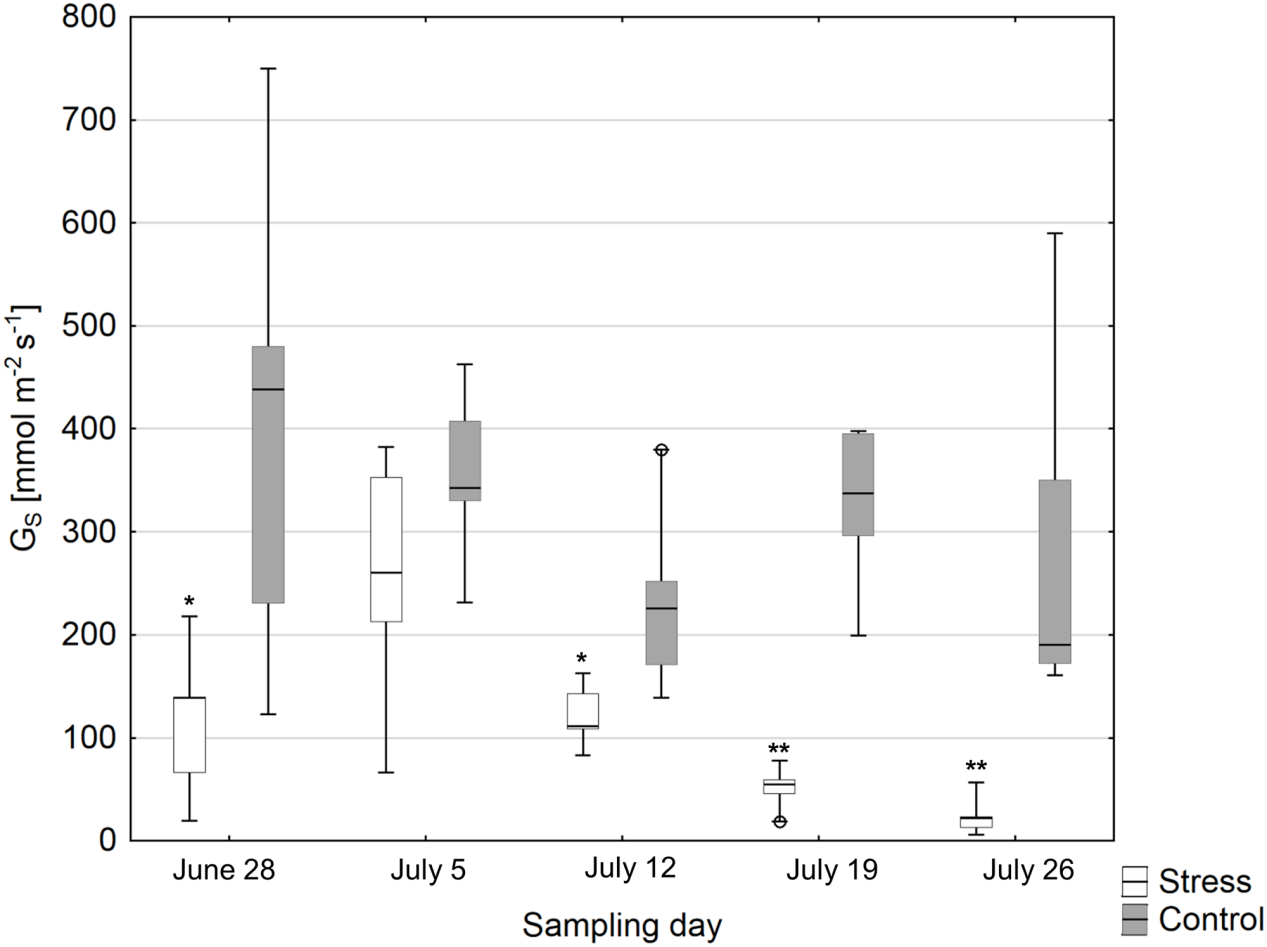
Box plots of stomatal conductance (G_*S*_) measured around noon of the drought stress and control group for the different sampling days. Asterisks indicate significant differences between the groups on each of the sampling days (*p<0.05, **p<0.01).

Stomatal conductance (*G*_*S*_) measured around noon of the plants selected for RNA-seq was significantly different between the drought and control group over all sampling days (p<0.0001). Mean *G*_*S*_ was lower in the drought stress plants than the control plants on each sampling day (Fig 1; difference significant except for July 5).

Genotyping of the ten selected samples for RNA-seq with microsatellite markers revealed a mean observed heterozygosity of 0.608, a mean expected heterozygosity of 0.507, and a mean fixation index of -0.076. There were no statistically significant differences for the genetic diversity indices between the drought stress and control group. All individuals had a unique multilocus genotype, hence no clones were selected.

### Sequencing output and *de novo* transcriptome assembly

Sequencing of the composite sample revealed 43,309,878 raw reads, which resulted in 41,186,808 reads after quality trimming (S2 File). The reads were assembled into 44,868 contigs with an average length of 764 bp, and a N50 contig length of 1,252 bp. After applying cd-hit-est to reduce the redundancy of the assembly, the number of contigs decreased to 44,335. Sequencing of the 50 samples of the drought stress experiment revealed a total of 2,324,791,074 reads after quality trimming. Individual libraries revealed 34,922,516 to 58,414,100 reads (mean 46,495,821.48 reads) (S2 File).

### Sequence annotation

**Fig 2:**
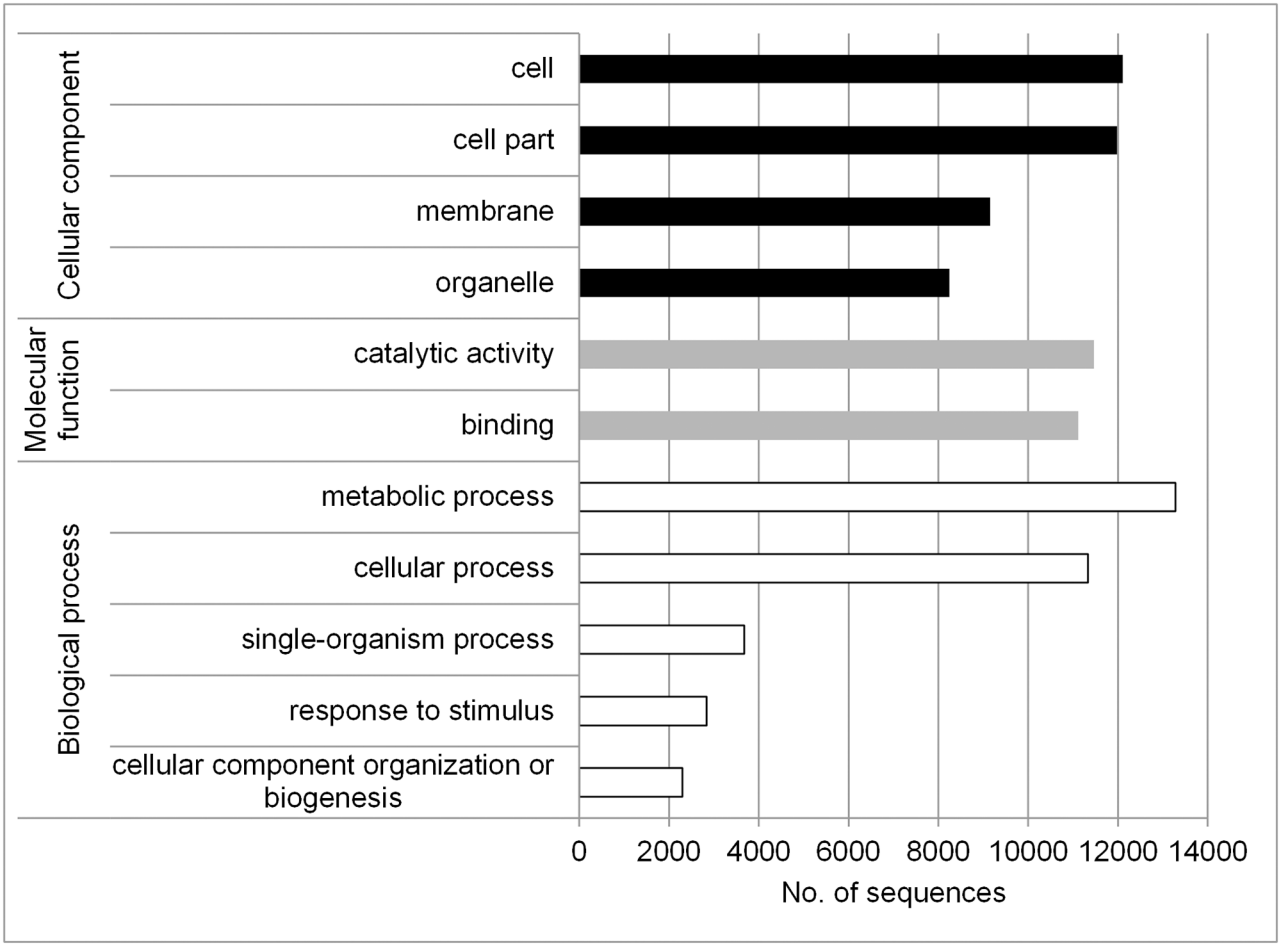
GO slim term distribution in the transcriptome.

BLAST results were obtained for 64.6% of all contigs. Although, an E-value cut-off of 1e^-3^ was chosen, most BLAST results were supported by much lower E-values. The complete annotation file can be found in the S3 File. The five species, which gave the highest number of BLAST hits were *Vitis vinifera*, *Citrus sinensis*, *Malus domestica*, *Theobroma cacao*, and *Populus trichocarpa*. GO terms were successfully assigned to 75.3% of the sequences with BLAST hits. GO slim terms with the highest abundance for cellular component, molecular function, and biological process terms were “cell”, “catalytic activity”, and “metabolic process” (Fig 2).

### Identification of differential gene expression

**Fig 3:**
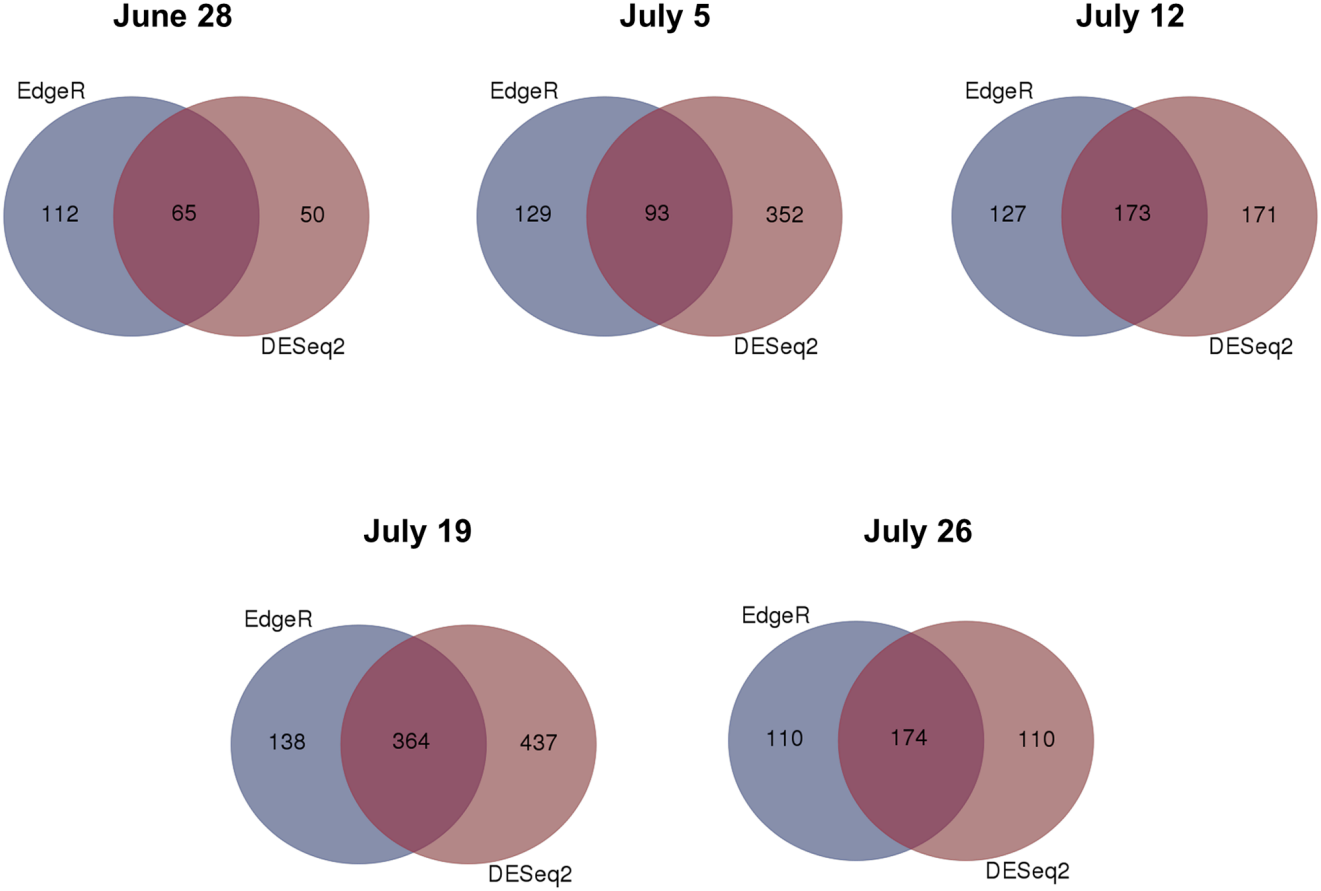
Venn diagrams of the number of DEGs between drought and control group for the different sampling days revealed by edgeR and DESeq2. Venn diagrams were prepared using the online tool provided by VIB and Ghent University (http://bioinformatics.psb.ugent.be/webtools/Venn/).

**Fig 4:**
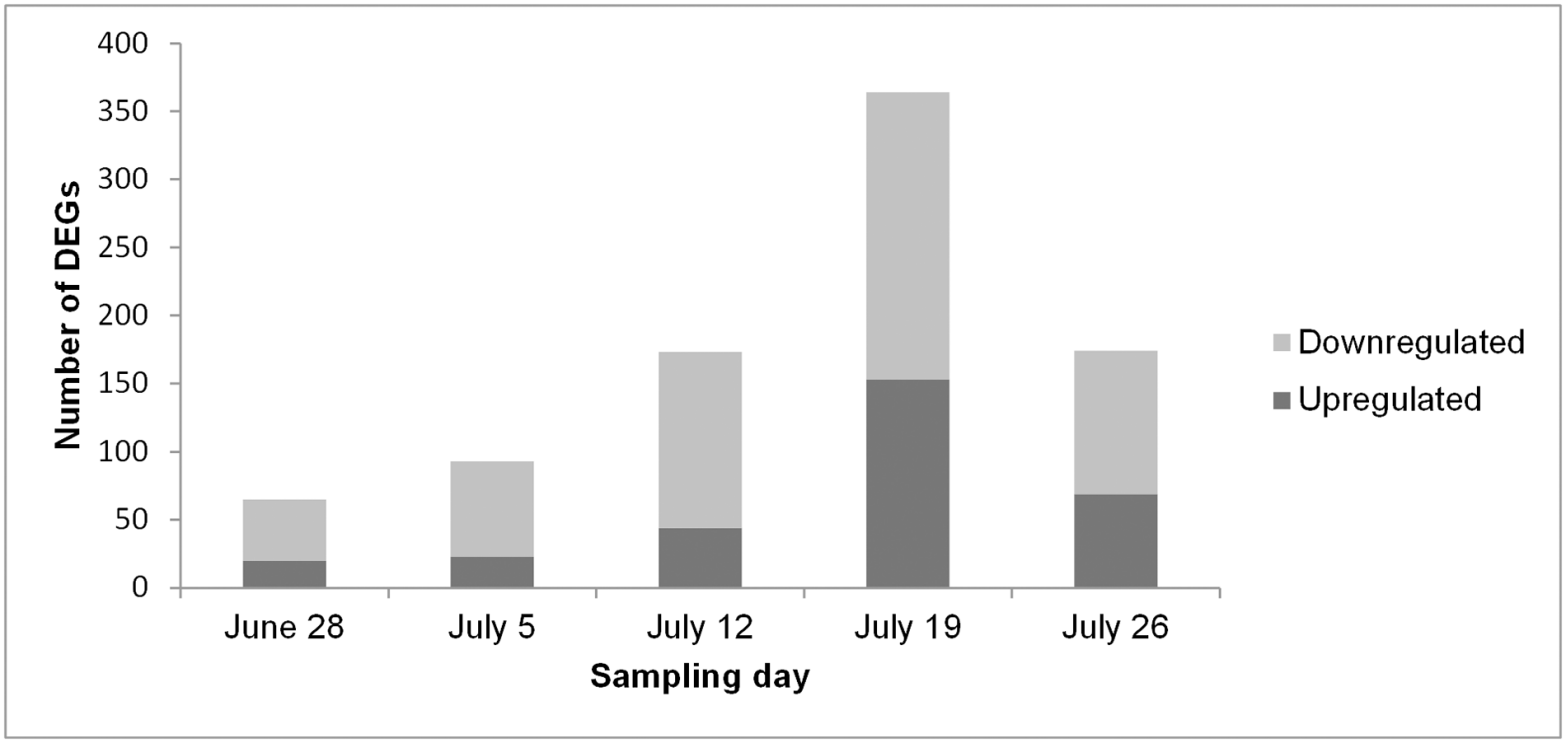
Number of DEGs on the different sampling days.

**Fig 5:**
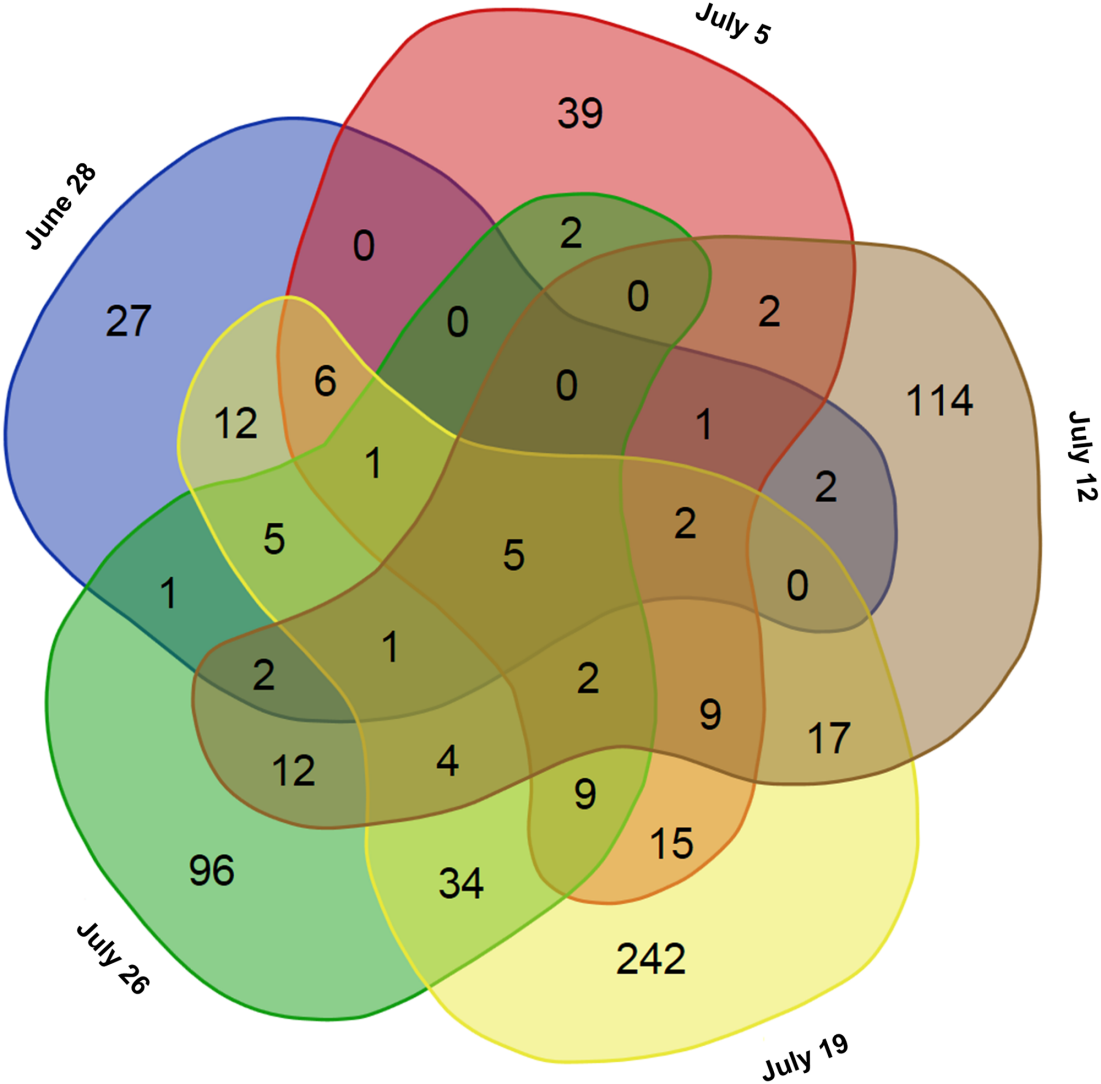
Venn diagrams for DEGs over all sampling days. The Venn diagram was prepared using the online tool provided by VIB and Ghent University (http://bioinformatics.psb.ugent.be/webtools/Venn/).

**Fig 6:**
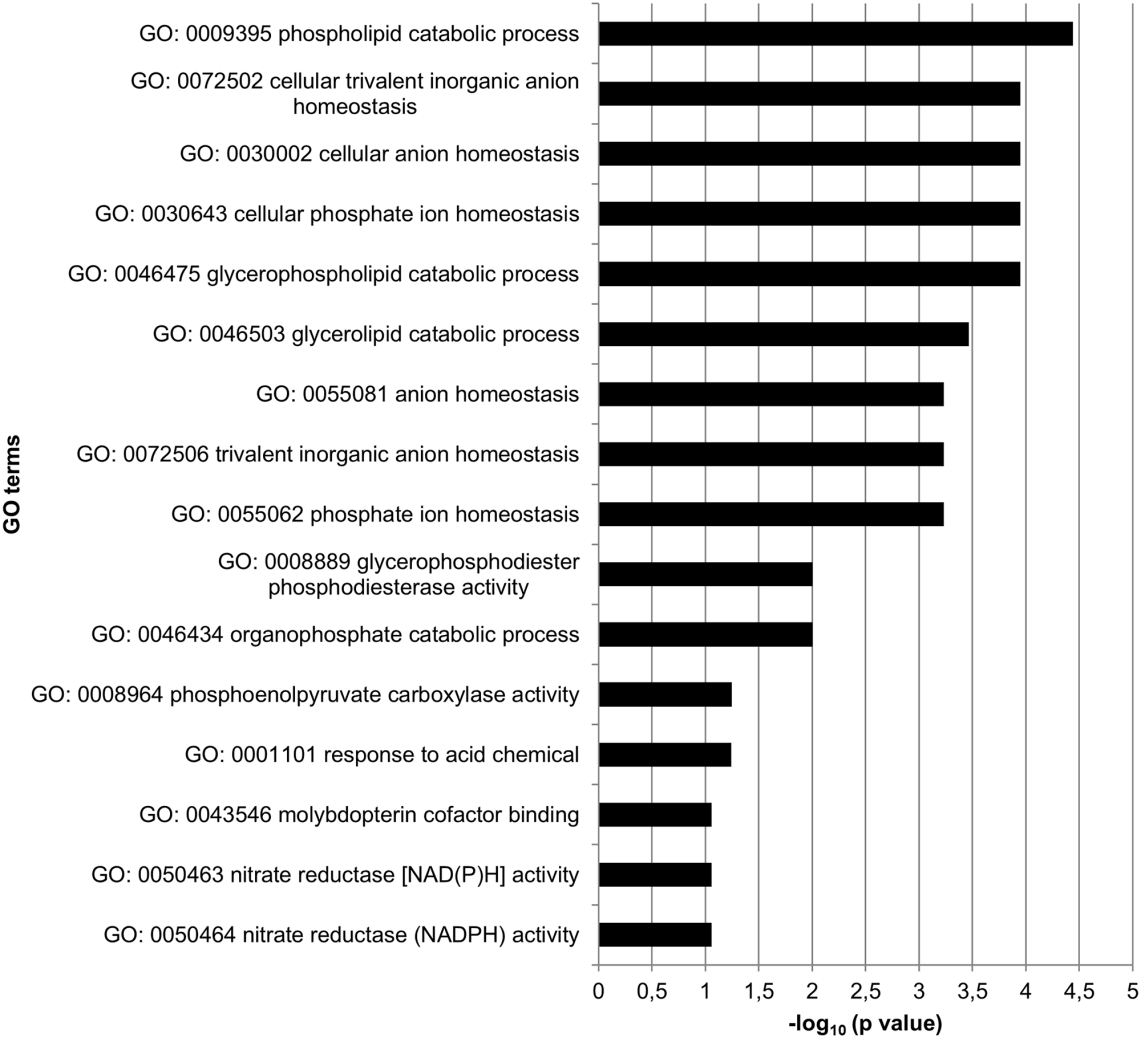
GO terms significantly enriched in upregulated DEGs compared to the reference gene set (total set of sequences with assigned GO terms) over all sampling days.

**Fig 7:**
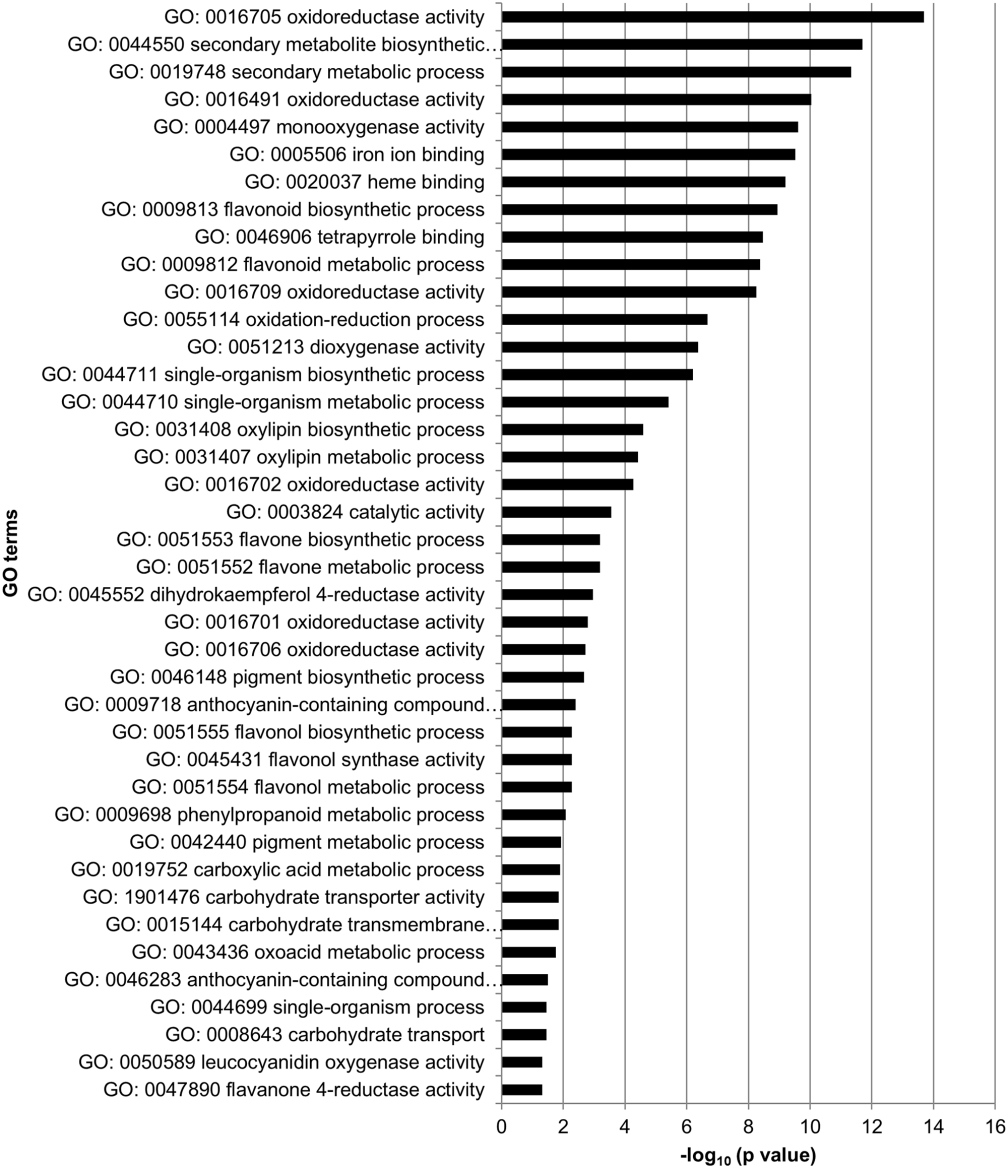
GO terms significantly enriched in downregulated DEGs compared to the reference gene set (total set of sequences with assigned GO terms) over all sampling days.

Significantly DEGs between the drought stress and control group were detected for all analyzed sampling days with both applied programs (edgeR and DESeq2) (S4 File), whereby DESeq2 detected more DEGs than edgeR on three of the five days (Fig 3). Nevertheless, many genes were overlapping between the two programs. Over all five sampling days 662 different genes were found to be differentially expressed among the stress and control group (only genes, which were revealed by both, edgeR and DESeq2) (S5 File). Thereby, the number of DEGs varied among sampling days ranging from 65 on June 28 to 364 on July 19 (Fig 4). More genes were downregulated than upregulated in the stress group on each sampling day. Gene expression was relatively specific to the respective sampling date (Fig 5) with 41.5% (June 28) to 66.5% (July 19) of genes exclusively expressed on the considered day. Only five genes (*protein yls9-like* (contig_2897), *UDP-glycosyltransferase74b1-like* (contig_1957), *receptor-like protein 12* (contig_21713), *probable lrr receptor-like serine threonine-protein kinase at4g36180-like* (contig_11937), and *protein p21-like* (contig_6745)) were significantly differentially expressed between the stress and control group on all five sampling days. All of these genes were downregulated in the stressed plants. GO term enrichment was statistically significant for the 662 unique DEGs, whereby GO terms were overrepresented in the set of DEGs compared to the total set of transcripts. Enriched GO terms with highest significance were “phospholipid catabolic process” (GO: 0009395), “glycerophospholipid catabolic process” (GO: 0046475), “cellular phosphate ion homeostasis” (GO: 0030643), “cellular anion homeostasis” (GO: 0030002), and “cellular trivalent inorganic anion homeostasis” (GO: 0072502) in the upregulated DEGs (Fig 6), and the GO terms “oxidoreductase activity” (GO: 0016705), “secondary metabolite biosynthetic process” (GO: 0044550), and “secondary metabolic process” (GO: 0019748) were most significantly enriched in the downregulated DEGs (Fig 7).

### Quantitative real-time PCR

**Fig 8:**
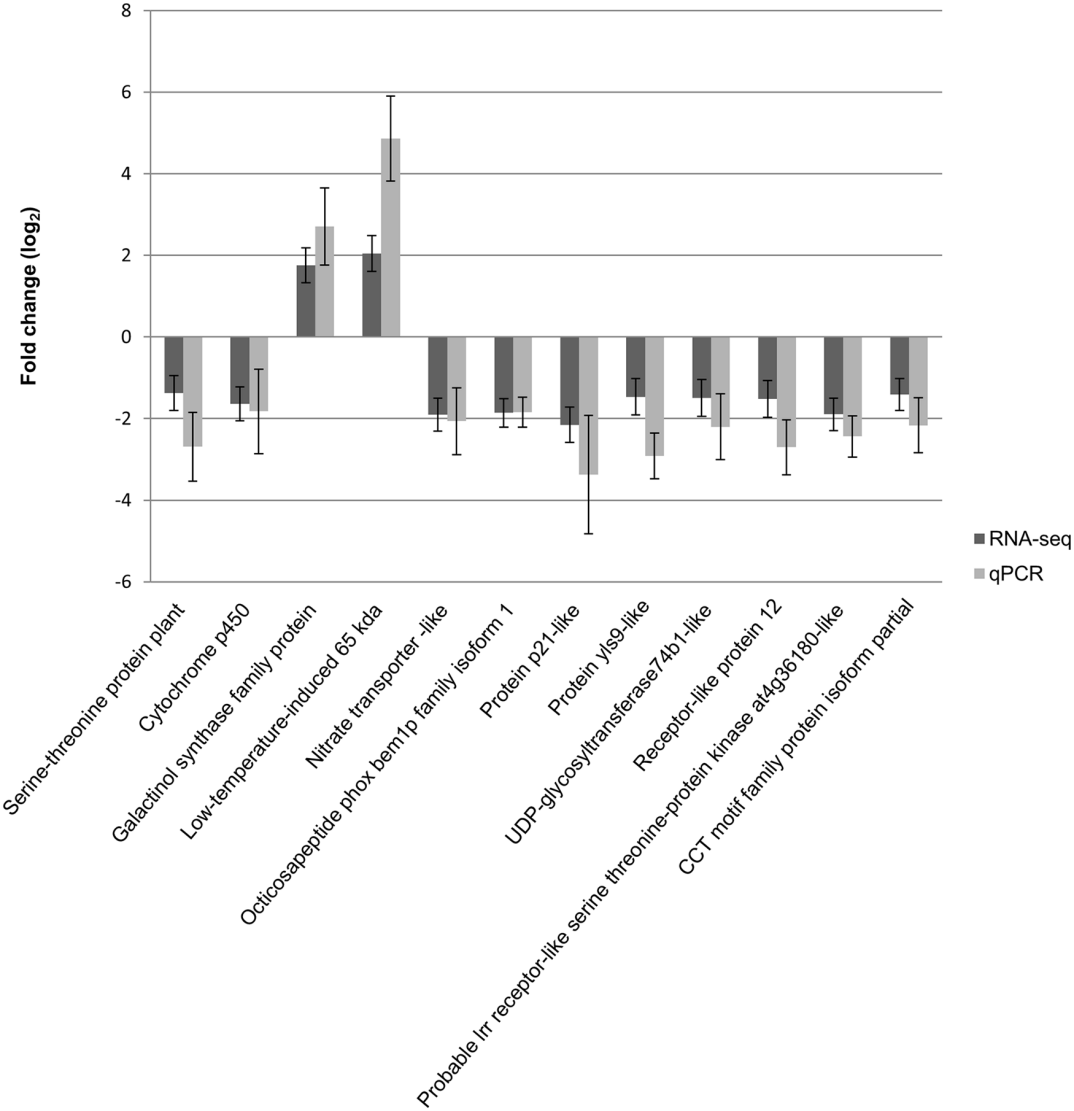
Comparison of expression patterns between RNA-seq and qPCR for selected transcripts. Bars indicate mean log_2_ fold change and whiskers indicate standard errors.

Eleven of the twelve selected genes showed expression profiles similar to those observed in the RNA-seq experiment (Fig 8). The genes *Galactinol synthase family protein*, and *Low-temperature-induced 65 kda* were upregulated in the stress group, while the genes *Nitrate transporter-like*, *Octicosapeptide phox bem1p family isoform 1*, *Protein p21-like*, *CCT motif family protein isoform partial*, *Probable lrr receptor-like serine threonine-protein kinase at4g36180-like*, *Receptor-like protein 12*, *UDP-glycosyltransferase74b1-like*, *Protein yls9-like* and *Serine-threonine protein plant* were downregulated in this group. Only the expression level of the gene *Cytochrome p450* was not statistically significant between the stress and control group contrary to the RNA-seq data. The expression levels, however, became significantly different between the two groups for this gene, after exclusion of the samples not used in the RNA-seq experiment.

## Discussion

### Drought stress experiment and SSR genotyping

Lower means of stomatal conductance measured at noon (*G*_s_) between the stress and control group on four of the five measuring days indicate that the drought treatment negatively affected leaf water status and gas exchange regulation. However, *G*_*s*_ was also dependent on weather conditions, notably vapor pressure deficit (VPD), which varied over the course of the experiment. The drought-exposed plants also developed a number of morphological, anatomical and other physiological modifications to water shortage (reduced aboveground productivity and root length, smaller leaf areas, reduced xylem hydraulic conductivity, smaller vessel diameters, higher embolism resistance), which distinguished them from the control plants [20, 38, 39].

SSR genotyping was used to characterize the neutral genetic structure of the ten selected individuals for RNA-seq. The analysis revealed observed and expected heterozygosities similar to those revealed by other studies in European beech (e.g., [25, 40, 41]. The genetic diversity indices were not different between the control and stress group. All individuals showed unique multilocus genotypes, hence no clones were selected for the analysis and the ten individuals represent real biological replicates.

### *De novo* transcriptome assembly and sequence annotation

The *de novo* transcriptome assembly was based on a normalized composite sample comprising all samples of the experiment to maximize gene discovery. The normalization step reduces high abundance transcripts and equalizes transcript concentrations. Hence, the redundancy of the cDNA library is reduced, which increases the efficiency of sequencing and rare gene discovery. In total, 41,186,808 high quality sequencing reads were assembled into 44,868 contigs. The average length of 764 bp and a N50 contig length of 1,252 bp are comparable to the results of *de novo* transcriptome assemblies in other forest tree species [19, 42, 43]. The usage of the program cd-hit-est [28] to reduce the redundancy of the assembly only slightly decreased the number of contigs (to 44,335). This shows that the assembly algorithm implemented in the CLC Genomics workbench and the normalization of the cDNA library resulted in a low redundancy *de novo* transcriptome assembly. This is in line with studies, which identified CLC as one of the leading assemblers producing low redundant assemblies [44, 45].

BLAST results were obtained for 64.6% of all contigs, whereby the five woody taxa *Vitis vinifera*, *Citrus sinensis*, *Malus domestica*, *Theobroma cacao*, and *Populus trichocarpa* were the species, which gave the highest number of BLAST hits. Although many transcripts were not functionally annotated, this study provides more than 28,500 annotated transcripts, which can directly be used for further research in European beech. The majority of the unannotated transcripts may be due to the lack of a reference genome of *F*. *sylvatica*, but the data set may also include some new beech-specific transcripts, since pooling and normalization of samples for *de novo* assembly should have enhanced the power of gene detection. GO-slim mapping was used to get an overview of the GO-term distribution over the whole transcriptome (based on the composite sample). The GO-terms with the highest abundance in the three categories “cellular component”, “molecular function”, and “biological process” were similar to the results of [19], who investigated the beech transcriptome related to dormancy regulation.

### Identification of differential gene expression

For the identification of differentially expressed genes the two widely used tools edgeR and DESeq2 [33, 34] were used. Both tools were found to perform better than other tools when the number of biological replicates was lower than 12 (as in the present study) [46]. Since different studies showed that the number of significantly DEGs can differ between edgeR and DESeq2 (or DESeq the previous version of DESeq2) [46–48], we decided to apply both of them. Indeed, both programs revealed a different number of DEGs for the different sampling days, whereby DESeq2 detected more DEGs than edgeR on three of the five days. Nevertheless, many DEGs were revealed by both programs. Only these overlapping genes were regarded as differentially expressed between the drought stress and control group in this study. Hence, the reported number of DEGs might be a rather conservative estimate.

Interestingly, the number of DEGs differed among sampling days. It increased from the first to the fourth sampling day and decreased on the last sampling day. The same pattern was observed for GO term distribution (S6 File), whereas the GO terms “ion binding” (GO: 0043167), “organic cyclic compound binding” (GO: 0097159), “heterocyclic compound binding” (GO: 1901363), and “transferase activity” (GO: 0016740) were among the most frequent ones on all sampling days. Zhang et al. [49] found a correlation of the number of drought-regulated genes with drought stress intensity and duration. This might also partially explain the observed pattern in the present study. While drought stress intensity (expressed by the SWC of the pots) was kept relatively constant during the experiment, the duration of drought stress increased from the first to the last sampling day. This, however, cannot explain the decrease of the number of DEGs on the last sampling day. The difference in gene expression in course of the experiment may rather be explained by a combination of both, the duration of drought stress treatment, and some variation in drought stress levels due to weather conditions resulting in different VPDs as indicated by the stomatal conductance. Furthermore, samples were not taken at exactly the same time on the different sampling days, which may additionally have induced some variation in drought-induced gene expression.

Only five genes (*protein yls9-like* (contig_2897), *UDP-glycosyltransferase74b1-like* (contig_1957), *receptor-like protein 12* (contig_21713), *probable lrr receptor-like serine threonine-protein kinase at4g36180-like* (contig_11937), and *protein p21-like* (contig_6745)) were significantly differentially expressed between the stress and control group on all five sampling days. All of these genes are assumed to be involved in some kind of stress resistance. For instance, a *UDP-glycosyltransferase74b1-like* gene was shown to be upregulated under drought stress in tobacco roots [50]. UPD-glycosyltransferases are the most common enzymes catalyzing glycosylation, a process required in a number of biological processes during plant growth and development, and can also be involved in abiotic stress adaptation [51]. The *YLS9* gene is related to leaf senescence in *Arabidopsis thaliana*, an important process for plant survival and adaptation to unfavorable environmental conditions [52]. Furthermore, the sequence of *protein YLS9-like* contains a *LEA* domain. LEA proteins play crucial roles in cellular dehydration tolerance [53]. Receptor-like proteins (RLPs) are cell surface receptors that typically obtain a leucine-rich repeat domain [54]. This kind of domain was also found in the sequences of *receptor-like protein 12* and *probable lrr receptor-like serine threonine-protein kinase at4g36180-like* in the present study. RLPs and receptor-like protein kinases are involved in different biological processes such as plant development, disease resistance, and stress tolerance [55, 56]. Protein P21 is a member of the PR-5 family, the members of which are also known as thaumatin-like proteins (TLPs), and are responsive to biotic and abiotic stress [57, 58]. Interestingly, all of these five genes in the present study were downregulated in drought stress plants compared to the control group. Thus, their expression might regulate some downstream stress response genes/pathways. Li et al. [51] for example, showed that the expression of a *UPD-glycosyltransferase* influenced the expression of other stress-inducible genes. Downregulation of the gene enhanced drought tolerance in *Arabidopsis* seedlings, whereas on over-expression reduced drought tolerance. The opposite trend was detected for mature *Arabidopsis* plants [51]. Nevertheless, the function of the five genes in drought stress response in beech remains open in our study, but they are interesting candidates for further studies. The different expression of these genes between the stress and control group on each of the sampling days indicate an important role in stress tolerance in beech. Using the developed primers for qPCR, these genes can immediately be investigated in other beech populations or desiccation experiments. Furthermore, the genes are candidate genes for drought stress tolerance, which can be used in association studies.

Over all five sampling days, 662 different genes were found to be differentially expressed between the stress and control group. We performed an enrichment analysis for GO terms in all up- and downregulated DEGs separately. GO terms with highest significance in the upregulated DEGs were “phospholipid catabolic process” (GO: 0009395), “glycerophospholipid catabolic process” (GO: 0046475), “cellular phosphate ion homeostasis” (GO: 0030643), “cellular anion homeostasis” (GO: 0030002), and “cellular trivalent inorganic anion homeostasis” (GO: 0072502). Thus, mainly genes involved in lipid- and homeostasis-related processes were upregulated. Homeostasis signaling leads to stress tolerance in plants [59], and lipids are important membrane components. Changes in their composition may help to maintain membrane integrity under water stress [60]. In the downregulated DEGs most significantly enriched GO terms were “oxidoreductase activity” (GO: 0016705), “secondary metabolite biosynthetic process” (GO: 0044550), and “secondary metabolic process” (GO: 0019748). All these GO terms can be connected with oxidative stress. Drought stress can cause oxidative stress through an enhanced production of reactive oxygen species (ROS) [61]. At low levels, however, ROS may also function as components of the stress-signaling pathway, triggering defense and/or acclimation responses [61, 62]. Antioxidant enzymes have often been called “first line of defense” against oxidative stress, whereby the activity of these enzymes can be enhanced or repressed depending on species, genotype, and stress duration/severity [63–66].

Shinozaki and Yamaguchi-Shinozaki (2007) [67] classified products of drought stress-inducible genes in *Arabidopsis* into functional ones, involved in abiotic stress tolerance (e.g., water channels, LEA proteins, chaperones, detoxification enzymes), and regulatory ones, involved in stress-responsive gene expression (e.g., transcription factors, protein kinases, enzymes involved in phospholipid metabolism). The 662 DEGs between the stress and normally watered plants include many genes, which can be assigned to these groups and/or are common drought stress inducible genes (e.g., late embryogenesis abundant proteins, aquaporins, heat shock proteins, and protein kinases). Similar results were revealed by a recent study of the water stress-related transcriptome of *Quercus lobata* [68], a species from the same family as European beech (Fagaceae).

In general, the results of RNA-seq may not be representative for the whole exome, since genes with low expression levels may be missed, or some genes are expressed in unsampled tissue [69]. In the present study, we only sampled leaf material, hence, genes involved in drought stress response may be different in other plant tissues such as roots or xylem. Furthermore, one weakness of the study design is that we did not take samples at the very beginning of the drought stress treatment, and the plants had been subjected to drought before the start of the investigated drought experiment. Thus, genes involved in the first response to drought stress may not have been identified. Some acclimations to drought stress from the first drought treatment can also not be ruled out, since plants can develop a stress memory after repeated drought periods [70].

### Quantitative real-time PCR

Quantitative real-time PCR was used to confirm differential gene expression revealed by RNA-seq. For qPCR validation, samples from individuals used for RNA-seq and additional samples from the drought stress experiment not used for RNA-seq were investigated. Eleven of the twelve selected genes showed similar expression as in the RNA-seq experiment. This is in line with other studies, which revealed a high correlation between qPCR and RNA-seq data [71–73]. Only for one tested gene (*Cytochrome p450*), the expression pattern revealed by RNA-seq could not be confirmed by qPCR. Nevertheless, after exclusion of the samples not used in the RNA-seq experiment, a validation of the RNA-seq experiment was possible. Thus, the expression of this gene might be genotype-specific.

### Conclusions

In this study, we report more than 28,500 annotated transcripts, which can directly be used for adaptation research in *F*. *sylvatica*. In total, 662 DEGs were identified between the drought stress and control group. These genes are candidate genes for drought stress adaptation, and can, for instance, be further investigated in association studies. This study shows that gene expression can be specific for different time points during drought stress treatment. GO term enrichment revealed that mostly genes involved in lipid- and homeostasis-related processes were upregulated in the stress plants, whereas genes involved in oxidative stress response were downregulated. This is a first insight into the genomic drought stress response of European beech. Further research may unravel the mechanism of genomic drought stress adaptation in greater detail by investigating important mechanisms of gene regulation such as alternative splicing or epigenetic effects. Nevertheless, the establishment of a reference genome for *F*. *sylvatica* would be an important prerequisite for a deeper understanding of adaptation in this species.

## Supporting information

S1 File Genes with regarding primer sequences used for qPCR. All genes were amplified with an annealing temperature of 58°C. The contig numbers refer to the sequence description in the transcriptome assembly.
(PDF)

S2 File Overview over the quality and quantity of sequencing reads for each sample before and after trimming. (PDF)

S3 File Annotation for the complete set of contigs. (XLSX)

S4 File Lists of differentially expressed genes between the stress and control group for the different sampling days. (XLSX)

S5 File List of all 662 differentially expressed genes between the stress and control group over all sampling days. (XLSX)

S6 File GO term distribution for the different sampling days. (PDF)

## References

[1] IPCC. Climate Change 2014: Synthesis Report. Contribution of Working Groups I, II and III to the Fith Assessment Report of the Intergovernmental Panel on Climate Change [Core Writing Team, R.K. Pachauri and L.A. Meyer (eds.)] Geneva, Switzerland; 2014.

[2] Dulamsuren C, Hauck M, Kopp G, Ruff M, Leuschner C. European beech responds to climate change with growth decline at lower, and growth increase at higher elevations in the center of its distribution range (SW Germany). Trees. 2016;31(2): 673–86. doi: 10.1007/s00468-016-1499-x

[3] Knutzen F, Dulamsuren C, Meier IC, Leuschner C. Recent climate warming-related growth decline impairs European beech in the center of its distribution range. Ecosystems. 2017 doi: 10.1007/s10021-017-0128-x

[4] Köcher P, Gebauer T, Horna V, Leuschner C. Leaf water status and stem xylem flux in relation to soil drought in five temperate broad-leaved tree species with contrasting water use strategies. Annals of Forest Science. 2009;66(1): 101-. doi: 10.1051/forest/2008076

[5] Aranda I, Gil L, Pardos JA. Water relations and gas exchange in *Fagus sylvatica* L. and *Quercus petraea* (Mattuschka) Liebl. in a mixed stand at their southern limit of distribution in Europe. Trees-Struct Funct. 2000;14(6): 344–52. doi: 10.1007/s004680050229

[6] Leuschner C, Backes K, Hertel D, Schipka F, Schmitt U, Terborg O, et al Drought responses at leaf, stem and fine root levels of competitive *Fagus sylvatica* L. and *Quercus petraea* (Matt.) Liebl. trees in dry and wet years. Forest Ecol Manag. 2001;149(1–3): 33–46. doi: 10.1016/S0378-1127(00)00543-0

[7] Zimmermann J, Hauck M, Dulamsuren C, Leuschner C. Climate warming-related growth decline affects *Fagus sylvatica*, but not other broad-leaved tree species in Central European mixed forests. Ecosystems. 2015;18(4): 560–72. doi: 10.1007/s10021-015-9849-x

[8] Hacket-Pain AJ, Cavin L, Friend AD, Jump AS. Consistent limitation of growth by high temperature and low precipitation from range core to southern edge of European beech indicates widespread vulnerability to changing climate. European Journal of Forest Research. 2016;135(5): 897–909. doi: 10.1007/s10342-016-0982-7

[9] Geßler A, Keitel C, Kreuzwieser J, Matyssek R, Seiler W, Rennenberg H. Potential risks for European beech (*Fagus sylvatica* L.) in a changing climate. Trees. 2007;21(1): 1–11. doi: 10.1007/s00468-006-0107-x

[10] Schuldt B, Knutzen F, Delzon S, Jansen S, Müller-Haubold H, Burlett R, et al How adaptable is the hydraulic system of European beech in the face of climate change-related precipitation reduction? New Phytol. 2016;210(2): 443–58. doi: 10.1111/nph.13798 26720626 10.1111/nph.13798

[11] Peuke AD, Schraml C, Hartung W, Rennenberg H. Identification of drought-sensitive beech ecotypes by physiological parameters. New Phytologist. 2002;154(2): 373–87. doi: 10.1046/j.1469-8137.2002.00400.x 10.1046/j.1469-8137.2002.00400.x 33873420

[12] Meier IC, Leuschner C. Genotypic variation and phenotypic plasticity in the drought response of fine roots of European beech. Tree Physiol. 2008;28(2): 297–309. 18055440 10.1093/treephys/28.2.297

[13] Schall P, Lödige C, Beck M, Ammer C. Biomass allocation to roots and shoots is more sensitive to shade and drought in European beech than in Norway spruce seedlings. Forest Ecol Manag. 2012;266: 246–53. doi: 10.1016/j.foreco.2011.11.017

[14] Carsjens C, Nguyen Ngoc Q, Guzy J, Knutzen F, Meier IC, Müller M, et al Intra-specific variations in expression of stress-related genes in beech progenies are stronger than drought-induced responses. Tree Physiol. 2014;34(12): 1348–61. doi: 10.1093/treephys/tpu093 25430883 10.1093/treephys/tpu093

[15] Knutzen F, Meier IC, Leuschner C. Does reduced precipitation trigger physiological and morphological drought adaptations in European beech (*Fagus sylvatica* L.)? Comparing provenances across a precipitation gradient. Tree Physiol. 2015;35(9): 949–63. doi: 10.1093/treephys/tpv057 26209617 10.1093/treephys/tpv057

[16] Dounavi A, Netzer F, Celepirovic N, Ivanković M, Burger J, Figueroa AG, et al Genetic and physiological differences of European beech provenances (*F*. *sylvatica* L.) exposed to drought stress. Forest Ecol Manag. 2016;361: 226–36. doi: 10.1016/j.foreco.2015.11.014

[17] Aranda I, Cano FJ, Gasco A, Cochard H, Nardini A, Mancha JA, et al Variation in photosynthetic performance and hydraulic architecture across European beech (*Fagus sylvatica* L.) populations supports the case for local adaptation to water stress. Tree Physiol. 2015;35(1): 34–46. doi: 10.1093/treephys/tpu101 25536961 10.1093/treephys/tpu101

[18] Gallois A, Burrus M, Brown S. Evaluation of the nuclear DNA content and GC percent in four varieties of *Fagus sylvatica* L. Annals of Forest Science. 1999;56(7): 615–8. doi: 10.1051/forest:19990709

[19] Lesur I, Bechade A, Lalanne C, Klopp C, Noirot C, Leple JC, et al A unigene set for European beech (*Fagus sylvatica* L.) and its use to decipher the molecular mechanisms involved in dormancy regulation. Mol Ecol Resour. 2015;15(5): 1192–204. doi: 10.1111/1755-0998.12373 25594128 10.1111/1755-0998.12373

[20] Lübbe T, Schuldt B, Coners H, Leuschner C. Species diversity and identity effects on the water consumption of tree sapling assemblages under ample and limited water supply. Oikos. 2016;125(1): 86–97. doi: 10.1111/oik.02367

[21] Müller M, Seifert S, Finkeldey R. A candidate gene-based association study reveals SNPs significantly associated with bud burst in European beech (*Fagus sylvatica* L.). Tree Genet Genom. 2015;11(6): 116 doi: 10.1007/s11295-015-0943-1

[22] Pastorelli R, Smulders MJM, Van't Westende WPC, Vosman B, Giannini R, Vettori C, et al Characterization of microsatellite markers in *Fagus sylvatica* L. and *Fagus orientalis* Lipsky. Mol Ecol Notes. 2003;3(1): 76–8. doi: 10.1046/j.1471-8286.2003.00355.x

[23] Asuka Y, Tani N, Tsumura Y, Tomaru N. Development and characterization of microsatellite markers for *Fagus crenata* Blume. Mol Ecol Notes. 2004;4(1): 101–3. doi: 10.1046/j.1471-8286.2003.00583.x

[24] Durand J, Bodenes C, Chancerel E, Frigerio JM, Vendramin G, Sebastiani F, et al A fast and cost-effective approach to develop and map EST-SSR markers: oak as a case study. BMC Genomics. 2010;11: 570 doi: 10.1186/1471-2164-11-570 20950475 10.1186/1471-2164-11-570 PMC3091719

[25] Vornam B, Decarli N, Gailing O. Spatial distribution of genetic variation in a natural beech stand (*Fagus sylvatica* L.) based on microsatellite markers. Conserv Genet. 2004;5(4): 561–70. doi: 10.1023/B:COGE.0000041025.82917.ac

[26] Peakall R, Smouse PE. genalex 6: genetic analysis in Excel. Population genetic software for teaching and research. Mol Ecol Notes. 2006;6(1): 288–95. doi: 10.1111/j.1471-8286.2005.01155.x 10.1093/bioinformatics/bts460 PMC3463245 22820204

[27] Peakall R, Smouse PE. GenAlEx 6.5: genetic analysis in Excel. Population genetic software for teaching and research—an update. Bioinformatics. 2012;28(19): 2537–9. doi: 10.1093/bioinformatics/bts460 22820204 10.1093/bioinformatics/bts460 PMC3463245

[28] Huang Y, Niu B, Gao Y, Fu L, Li W. CD-HIT Suite: a web server for clustering and comparing biological sequences. Bioinformatics. 2010;26(5): 680–2. doi: 10.1093/bioinformatics/btq003 20053844 10.1093/bioinformatics/btq003 PMC2828112

[29] Conesa A, Götz S, Garcia-Gomez JM, Terol J, Talon M, Robles M. Blast2GO: a universal tool for annotation, visualization and analysis in functional genomics research. Bioinformatics. 2005;21(18): 3674–6. doi: 10.1093/bioinformatics/bti610 16081474 10.1093/bioinformatics/bti610

[30] Altschul SF, Gish W, Miller W, Myers EW, Lipman DJ. Basic local alignment search tool. Journal of Molecular Biology. 1990;215(3): 403–10. doi: 10.1016/S0022-2836(05)80360-2 2231712 10.1016/S0022-2836(05)80360-2

[31] Ashburner M, Ball CA, Blake JA, Botstein D, Butler H, Cherry JM, et al Gene ontology: tool for the unification of biology. The Gene Ontology Consortium. Nat Genet. 2000;25(1): 25–9. doi: 10.1038/75556 10802651 10.1038/75556 PMC3037419

[32] Conesa A, Götz S. Blast2GO: A comprehensive suite for functional analysis in plant genomics. Int J Plant Genomics. 2008;2008: 619832 doi: 10.1155/2008/619832 18483572 10.1155/2008/619832 PMC2375974

[33] Robinson MD, McCarthy DJ, Smyth GK. edgeR: a Bioconductor package for differential expression analysis of digital gene expression data. Bioinformatics. 2010;26(1): 139–40. doi: 10.1093/bioinformatics/btp616 19910308 10.1093/bioinformatics/btp616 PMC2796818

[34] Love MI, Huber W, Anders S. Moderated estimation of fold change and dispersion for RNA-seq data with DESeq2. Genome Biol. 2014;15(12): 550 doi: 10.1186/s13059-014-0550-8 25516281 10.1186/s13059-014-0550-8 PMC4302049

[35] Benjamini Y, Hochberg Y. Controlling the False Discovery Rate: A Practical and Powerful Approach to Multiple Testing. Journal of the Royal Statistical Society Series B (Methodological). 1995;57(1): 289–300.

[36] Ye J, Coulouris G, Zaretskaya I, Cutcutache I, Rozen S, Madden TL. Primer-BLAST: a tool to design target-specific primers for polymerase chain reaction. BMC Bioinformatics. 2012;13: 134 doi: 10.1186/1471-2105-13-134 22708584 10.1186/1471-2105-13-134 PMC3412702

[37] Olbrich M, Gerstner E, Welzl G, Fleischmann F, Osswald W, Bahnweg G, et al Quantification of mRNAs and housekeeping gene selection for quantitative real-time RT-PCR normalization in European beech (*Fagus sylvatica* L.) during abiotic and biotic stress. Z Naturforsch C. 2008;63(7–8): 574–82. 18811005 10.1515/znc-2008-7-819

[38] Lübbe T, Schuldt B, Leuschner C. Species identity and neighbor size surpass the impact of tree species diversity on productivity in experimental broad-leaved tree sapling assemblages under dry and moist conditions. Front Plant Sci. 2015;6: 857 doi: 10.3389/fpls.2015.00857 26579136 10.3389/fpls.2015.00857 PMC4620412

[39] Lübbe T, Schuldt B, Leuschner C. Acclimation of leaf water status and stem hydraulics to drought and tree neighbourhood: alternative strategies among the saplings of five temperate deciduous tree species. Tree Physiol. 2016. 10.1093/treephys/tpw095 27881798

[40] Rajendra KC, Seifert S, Prinz K, Gailing O, Finkeldey R. Subtle human impacts on neutral genetic diversity and spatial patterns of genetic variation in European beech (*Fagus sylvatica*). Forest Ecol Manag. 2014;319: 138–49. doi: 10.1016/j.foreco.2014.02.003

[41] Bilela S, Dounavi A, Fussi B, Konnert M, Holst J, Mayer H, et al Natural regeneration of *Fagus sylvatica* L. adapts with maturation to warmer and drier microclimatic conditions. Forest Ecol Manag. 2012;275: 60–7. doi: 10.1016/j.foreco.2012.03.009

[42] Torre S, Tattini M, Brunetti C, Fineschi S, Fini A, Ferrini F, et al RNA-seq analysis of *Quercus pubescens* Leaves: *de novo* transcriptome assembly, annotation and functional markers development. PLoS One. 2014;9(11): e112487 doi: 10.1371/journal.pone.0112487 25393112 10.1371/journal.pone.0112487 PMC4231058

[43] Lane T, Best T, Zembower N, Davitt J, Henry N, Xu Y, et al The green ash transcriptome and identification of genes responding to abiotic and biotic stresses. BMC Genomics. 2016;17: 702 doi: 10.1186/s12864-016-3052-0 27589953 10.1186/s12864-016-3052-0 PMC5009568

[44] Kumar S, Blaxter ML. Comparing *de novo* assemblers for 454 transcriptome data. BMC Genomics. 2010;11: 571 doi: 10.1186/1471-2164-11-571 20950480 10.1186/1471-2164-11-571 PMC3091720

[45] Honaas LA, Wafula EK, Wickett NJ, Der JP, Zhang Y, Edger PP, et al Selecting superior *de novo* transcriptome assemblies: lessons learned by leveraging the best plant genome. PLoS One. 2016;11(1): e0146062 doi: 10.1371/journal.pone.0146062 26731733 10.1371/journal.pone.0146062 PMC4701411

[46] Schurch NJ, Schofield P, Gierlinski M, Cole C, Sherstnev A, Singh V, et al How many biological replicates are needed in an RNA-seq experiment and which differential expression tool should you use? RNA. 2016;22(6): 839–51. doi: 10.1261/rna.053959.115 27022035 10.1261/rna.053959.115 PMC4878611

[47] Robles JA, Qureshi SE, Stephen SJ, Wilson SR, Burden CJ, Taylor JM. Efficient experimental design and analysis strategies for the detection of differential expression using RNA-Sequencing. BMC Genomics. 2012;13 doi: 10.1186/1471-2164-13-484 10.1186/1471-2164-13-484 PMC3560154 22985019

[48] Seyednasrollah F, Laiho A, Elo LL. Comparison of software packages for detecting differential expression in RNA-seq studies. Brief Bioinform. 2015;16(1): 59–70. doi: 10.1093/bib/bbt086 24300110 10.1093/bib/bbt086 PMC4293378

[49] Zhang JY, Cruz DECMH, Torres-Jerez I, Kang Y, Allen SN, Huhman DV, et al Global reprogramming of transcription and metabolism in *Medicago truncatula* during progressive drought and after rewatering. Plant Cell Environ. 2014;37(11): 2553–76. doi: 10.1111/pce.12328 24661137 10.1111/pce.12328 PMC4260174

[50] Rabara RC, Tripathi P, Reese RN, Rushton DL, Alexander D, Timko MP, et al Tobacco drought stress responses reveal new targets for Solanaceae crop improvement. BMC Genomics. 2015;16: 484 doi: 10.1186/s12864-015-1575-4 26123791 10.1186/s12864-015-1575-4 PMC4485875

[51] Li YJ, Wang B, Dong RR, Hou BK. *AtUGT76C2*, an *Arabidopsis* cytokinin glycosyltransferase is involved in drought stress adaptation. Plant Sci. 2015;236: 157–67. doi: 10.1016/j.plantsci.2015.04.002 26025529 10.1016/j.plantsci.2015.04.002

[52] Yoshida S, Ito M, Nishida I, Watanabe A. Isolation and RNA gel blot analysis of genes that could serve as potential molecular markers for leaf senescence in *Arabidopsis thaliana*. Plant Cell Physiol. 2001;42(2): 170–8. doi: 10.1093/pcp/pce021 11230571 10.1093/pcp/pce021

[53] Hincha DK, Thalhammer A. LEA proteins: IDPs with versatile functions in cellular dehydration tolerance. Biochem Soc Trans. 2012;40(5): 1000–3. doi: 10.1042/BST20120109 22988854 10.1042/BST20120109

[54] Wang G, Ellendorff U, Kemp B, Mansfield JW, Forsyth A, Mitchell K, et al A genome-wide functional investigation into the roles of receptor-like proteins in *Arabidopsis*. Plant Physiol. 2008;147(2): 503–17. doi: 10.1104/pp.108.119487 18434605 10.1104/pp.108.119487 PMC2409048

[55] Wu J, Liu Z, Zhang Z, Lv Y, Yang N, Zhang G, et al Transcriptional regulation of receptor-like protein genes by environmental stresses and hormones and their overexpression activities in *Arabidopsis thaliana*. J Exp Bot. 2016;67(11): 3339–51. doi: 10.1093/jxb/erw152 27099374 10.1093/jxb/erw152 PMC4892725

[56] Morris ER, Walker JC. Receptor-like protein kinases: the keys to response. Current Opinion in Plant Biology. 2003;6(4): 339–42. doi: 10.1016/s1369-5266(03)00055-4 12873528 10.1016/s1369-5266(03)00055-4

[57] Petre B, Major I, Rouhier N, Duplessis S. Genome-wide analysis of eukaryote thaumatin-like proteins (TLPs) with an emphasis on poplar. BMC Plant Biol. 2011;11: 33 doi: 10.1186/1471-2229-11-33 21324123 10.1186/1471-2229-11-33 PMC3048497

[58] Pechanova O, Pechan T. Maize-pathogen interactions: an ongoing combat from a proteomics perspective. Int J Mol Sci. 2015;16(12): 28429–48. doi: 10.3390/ijms161226106 26633370 10.3390/ijms161226106 PMC4691053

[59] Zhu JK. Salt and drought stress signal transduction in plants. Annu Rev Plant Biol. 2002;53: 247–73. doi: 10.1146/annurev.arplant.53.091401.143329 12221975 10.1146/annurev.arplant.53.091401.143329 PMC3128348

[60] Gigon A, Matos AR, Laffray D, Zuily-Fodil Y, Pham-Thi AT. Effect of drought stress on lipid metabolism in the leaves of Arabidopsis thaliana (ecotype Columbia). Ann Bot. 2004;94(3): 345–51. doi: 10.1093/aob/mch150 15277243 10.1093/aob/mch150 PMC4242175

[61] Cruz de Carvalho MH. Drought stress and reactive oxygen species: production, scavenging and signaling. Plant Signaling & Behavior. 2008;3(3): 156–65. 19513210 10.4161/psb.3.3.5536 PMC2634109

[62] Dat J, Vandenabeele S, Vranova E, Van Montagu M, Inze D, Van Breusegem F. Dual action of the active oxygen species during plant stress responses. Cell Mol Life Sci. 2000;57(5): 779–95. doi: 10.1007/s000180050041 10892343 10.1007/s000180050041 PMC11147059

[63] Liu C, Liu Y, Guo K, Fan D, Li G, Zheng Y, et al Effect of drought on pigments, osmotic adjustment and antioxidant enzymes in six woody plant species in karst habitats of southwestern China. Environmental and Experimental Botany. 2011;71(2): 174–83. doi: 10.1016/j.envexpbot.2010.11.012

[64] Grene R. Oxidative stress and acclimation mechanisms in plants. *Arabidopsis* Book. 2002;1: e0036 doi: 10.1199/tab.0036.1 22303206 10.1199/tab.0036.1 PMC3243402

[65] Fracasso A, Trindade LM, Amaducci S. Drought stress tolerance strategies revealed by RNA-Seq in two sorghum genotypes with contrasting WUE. BMC Plant Biol. 2016;16(1): 115 doi: 10.1186/s12870-016-0800-x 27208977 10.1186/s12870-016-0800-x PMC4875703

[66] Schwanz P, Polle A. Differential stress responses of antioxidative systems to drought in pendunculate oak (Quercus robur) and maritime pine (Pinus pinaster) grown under high CO2 concentrations. Journal of Experimental Botany. 2001;52(354): 133–43. doi: 10.1093/jexbot/52.354.133 11181722

[67] Shinozaki K, Yamaguchi-Shinozaki K. Gene networks involved in drought stress response and tolerance. J Exp Bot. 2007;58(2): 221–7. doi: 10.1093/jxb/erl164 17075077 10.1093/jxb/erl164

[68] Gugger PF, Penaloza-Ramirez JM, Wright JW, Sork VL. Whole-transcriptome response to water stress in a California endemic oak, *Quercus lobata*. Tree Physiol. 2017;37(5): 632–44. doi: 10.1093/treephys/tpw122 28008082 10.1093/treephys/tpw122

[69] Hoban S, Kelley JL, Lotterhos KE, Antolin MF, Bradburd G, Lowry DB, et al Finding the genomic basis of local adaptation: pitfalls, practical solutions, and future directions. Am Nat. 2016;188(4): 379–97. doi: 10.1086/688018 27622873 10.1086/688018 PMC5457800

[70] Fleta-Soriano E, Munne-Bosch S. Stress memory and the inevitable effects of drought: a physiological perspective. Front Plant Sci. 2016;7: 143 doi: 10.3389/fpls.2016.00143 26913046 10.3389/fpls.2016.00143 PMC4753297

[71] Ueno S, Klopp C, Leple JC, Derory J, Noirot C, Leger V, et al Transcriptional profiling of bud dormancy induction and release in oak by next-generation sequencing. BMC Genomics. 2013;14: 236 doi: 10.1186/1471-2164-14-236 23575249 10.1186/1471-2164-14-236 PMC3639946

[72] Garg R, Shankar R, Thakkar B, Kudapa H, Krishnamurthy L, Mantri N, et al Transcriptome analyses reveal genotype- and developmental stage-specific molecular responses to drought and salinity stresses in chickpea. Sci Rep. 2016;6: 19228 doi: 10.1038/srep19228 26759178 10.1038/srep19228 PMC4725360

[73] Shi Y, He M. Differential gene expression identified by RNA-Seq and qPCR in two sizes of pearl oyster (Pinctada fucata). Gene. 2014;538(2): 313–22. doi: 10.1016/j.gene.2014.01.031 24440293 10.1016/j.gene.2014.01.031

