# Supplementary material for: De novo transcriptome assembly and analysis of differential gene expression in response to drought in European beech
Source: PLoS One. 2017 Sep 5;12(9):e0184167. doi: 10.1371/journal.pone.0184167 (PMC5584803; doi:10.1371/journal.pone.0184167)
Supplement: S1 File — All genes were amplified with an annealing temperature of 58°C. The contig numbers refer to the sequence description in the transcriptome assembly. (PDF) [file pone.0184167.s001.pdf]

Gene	Contig no.	Forward primer sequence (5'-3')	Reverse primer sequence (5'-3')	Primer efficiency [%]
Cytochrome p450	Contig_13761	CAGCGGTAGAAGAATGTGCC	GTTCATCTGACGGGTTTGCA	100
Galactinol synthase family protein	Contig_6371	ATGGAGGTACACTGGGGAGG	GCACCAACAGAAGCCAATGT	105
Low-temperature-induced 65 kda	Contig_13090	CGGTTACTGGGACACTGAGT	TCATCACCAGGCCTCAACTT	101
Nitrate transporter -like	Contig_10659	CATGAAGGCGGGATCACTCT	CCTAAGGGTTGTGGGTGCAA	99
Octicosapeptide phox bem1p family isoform 1	Contig_5573	CTCTCAACAACAGCGTTGGC	CTGCCGGGTCGACCTTATTT	100
Protein p21-like	Contig_6745	CACATTTACGTGCCCTGGTG	AGCGTCTGCGCAACACTAAT	97
Serine-threonine protein plant	Contig_14505	CTTTCCCAGCCGACATAAGC	GTCAGAAGATTCCACGGTGC	97
Protein yls9-like	Contig_2897	GGGAGTCAGATAAGTGGGTGG	TGTCCGGTCAAAGCCAAGTT	96
UDP-glycosyltransferase74b1-like	Contig_1957	AATGGGTGGTGGCTAGAGTG	TGCTCACTTATCCTGCCCAA	100
Receptor-like protein 12	Contig_21713	TTTGCGGATCTTGGACCTCT	CACCTTCTTCCACGTTCGTC	94
Probable lrr receptor-like serine threonine-protein kinase at4g36180-like	Contig_11937	CTGTAACGCGCCAATGTCTG	TCTGGGACAATTCCGAGGGA	95
CCT motif family protein isoform partial	Contig_1104	CATGGTCAATCCCTGGCCTT	GACATGGGTAAGGACCACCA	95
