# Supplementary material for: De novo transcriptome assembly and analysis of differential gene expression in response to drought in European beech
Source: PLoS One. 2017 Sep 5;12(9):e0184167. doi: 10.1371/journal.pone.0184167 (PMC5584803; doi:10.1371/journal.pone.0184167)
Supplement: S2 File — (PDF) [file pone.0184167.s002.pdf]

Sample_ID	No. of reads before trimming	No. of reads after trimming	Mean PHRED-score before trimming	Mean PHRED-score after trimming
**Sample_M-13-5.7_R1**	44,623,168	44,117,574	34.9	35.8
**Sample_M-14-5.7_R1**	50,992,910	48,552,174	33.1	35.2
**Sample_M-21-5.7_R1**	47,356,660	46,737,498	34.4	35.5
**Sample_M-36-5.7_R1**	49,339,098	47,002,580	34.1	35.8
**Sample_M-43-5.7_R1**	46,668,714	46,323,498	35.2	35.9
**Sample_M-45-5.7_R1**	41,870,624	40,907,606	34.6	35.8
**Sample_M-46-5.7_R1**	46,489,636	45,829,022	34.3	35.5
**Sample_M-48-5.7_R1**	53,375,384	52,384,714	34.7	35.8
**Sample_M-51-5.7_R1**	43,802,330	43,409,632	34.3	35.4
**Sample_M-16-5.7_R1**	46,364,246	45,839,476	35.2	36.1
**Sample_M-13-12.7_R1**	41,054,422	40,513,112	34.7	35.7
**Sample_M-14-12.7_R1**	55,479,180	52,441,794	33.2	35.3
**Sample_M-16-12.7_R1**	49,735,274	49,283,462	35.4	36.2
**Sample_M-21-12.7_R1**	51,689,256	48,353,466	33.7	35.7
**Sample_M-36-12.7_R1**	48,788,538	48,292,778	34.5	35.5
**Sample_M-43-12.7_R1**	52,433,046	49,932,656	34	35.8
**Sample_M-45-12.7_R1**	53,104,546	52,425,566	34.8	35.8
**Sample_M-46-12.7_R1**	52,922,942	49,880,058	33.3	35.3
**Sample_M-48-12.7_R1**	48,989,730	48,401,164	34.6	35.6
**Sample_M-51-12.7_R1**	45,216,218	44,759,886	35	35.9
**Sample_M-13-19.7_R1**	41,356,318	40,853,662	35	36
**Sample_M-14-19.7_R1**	40,594,902	40,303,490	34.2	35.3
**Sample_M-16-19.7_R1**	44,677,018	41,336,976	33.7	36
**Sample_M-21-19.7_R1**	47,252,340	46,787,140	35.1	36
**Sample_M-36-19.7_R1**	47,460,264	44,522,060	33.7	35.8
**Sample_M-43-19.7_R1**	52,548,750	52,148,238	34.9	35.8
**Sample_M-45-19.7_R1**	49,333,812	46,350,628	33.4	35.5
**Sample_M-46-19.7_R1**	53,755,666	53,335,734	34.6	35.5
**Sample_M-48-19.7_R1**	52,268,774	51,921,888	35.2	36
**Sample_M-51-19.7_R1**	47,449,672	44,320,480	32.9	35.3
**Sample_M-13-26.7_R1**	44,444,056	43,965,736	34.8	35.8
**Sample_M-14-26.7_R1**	41,046,320	40,211,248	34.3	35.5
**Sample_M-16-26.7_R1**	46,246,094	45,919,658	35.4	36.2
**Sample_M-21-26.7_R1**	39,069,820	38,651,748	34.8	35.8
**Sample_M-36-26.7_R1**	38,592,754	38,256,240	34.5	35.6
**Sample_M-43-26.7_R1**	44,125,740	41,623,340	34.1	35.9
**Sample_M-45-26.7_R1**	50,409,884	49,992,034	35.1	35.9
**Sample_M-46-26.7_R1**	43,302,628	42,889,650	34.8	35.7
**Sample_M-48-26.7_R1**	47,348,584	46,694,530	34.6	35.7
**Sample_M-51-26.7_R1**	47,912,194	47,517,116	34.5	35.5
**Sample_M-13-28.6_R1**	59,485,248	58,414,100	34.6	35.7
**Sample_M-14-28.6_R1**	51,593,394	50,950,714	34.6	35.6
**Sample_M-16-28.6_R1**	47,567,016	45,642,818	34	35.7
**Sample_M-21-28.6_R1**	51,319,448	50,872,594	34.3	35.4

**Sample_M-36-28.6_R1**	47,584,788	46,959,900	34.6	35.7
**Sample_M-43-28.6_R1**	45,649,024	45,044,710	35	35.9
**Sample_M-45-28.6_R1**	44,935,416	44,497,572	34.9	35.8
**Sample_M-46-28.6_R1**	35,387,258	34,922,516	33.8	35.2
**Sample_M-48-28.6_R1**	51,185,830	50,804,380	35	35.8
**Sample_M-51-28.6_R1**	56,788,510	53,692,458	33.5	35.4
**Sample_Mischprobe-Mueller_R1**	43,309,878	41,186,808	33.8	35.6
