# Supplementary figures and images for: De novo transcriptome assembly and analysis of differential gene expression in response to drought in European beech
Source: PLoS One. 2017 Sep 5;12(9):e0184167. doi: 10.1371/journal.pone.0184167 (PMC5584803; doi:10.1371/journal.pone.0184167)

## Sampling day June 28

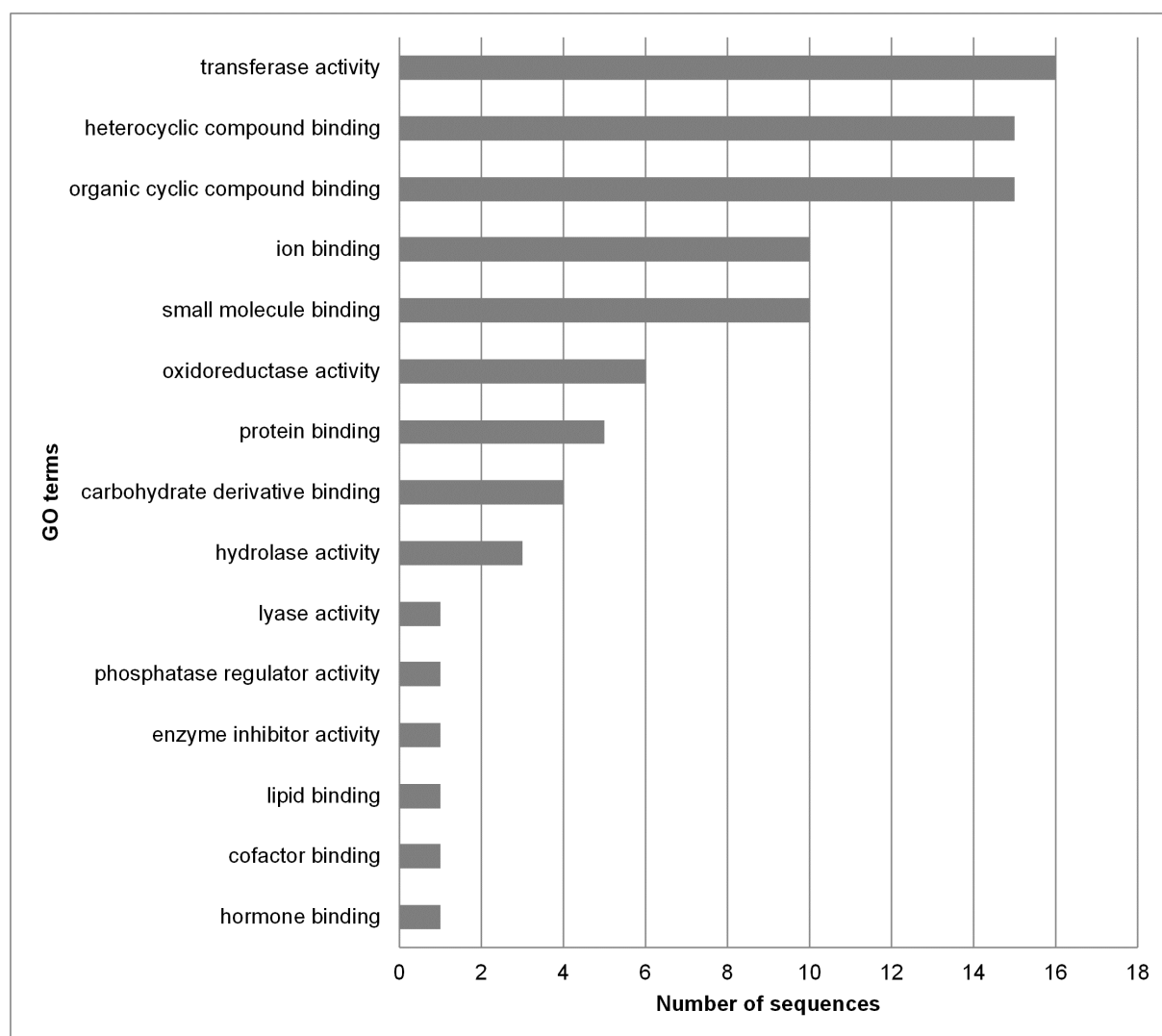

## Sampling day July 5

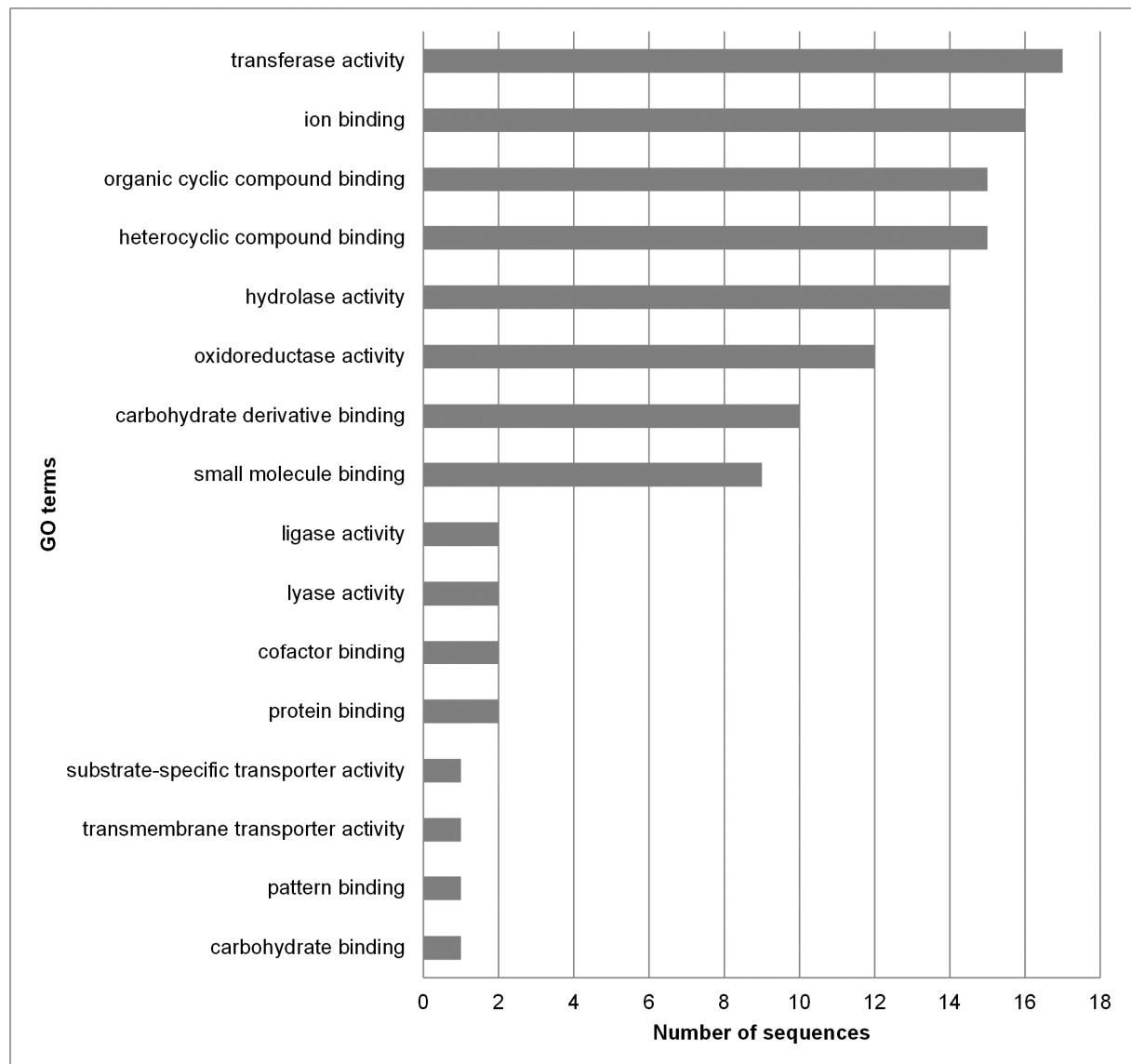

## Sampling day July 12

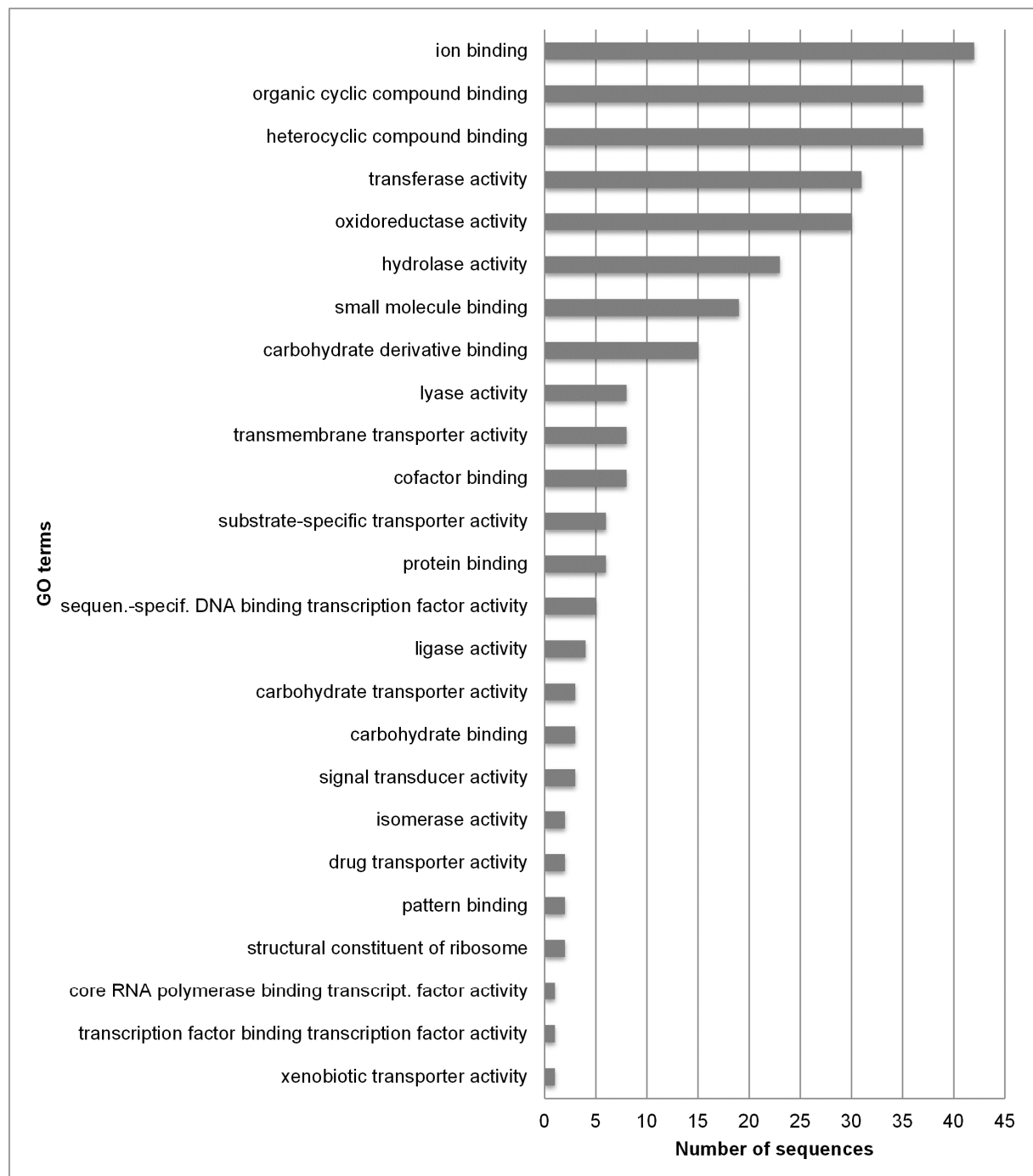

## Sampling day July 19

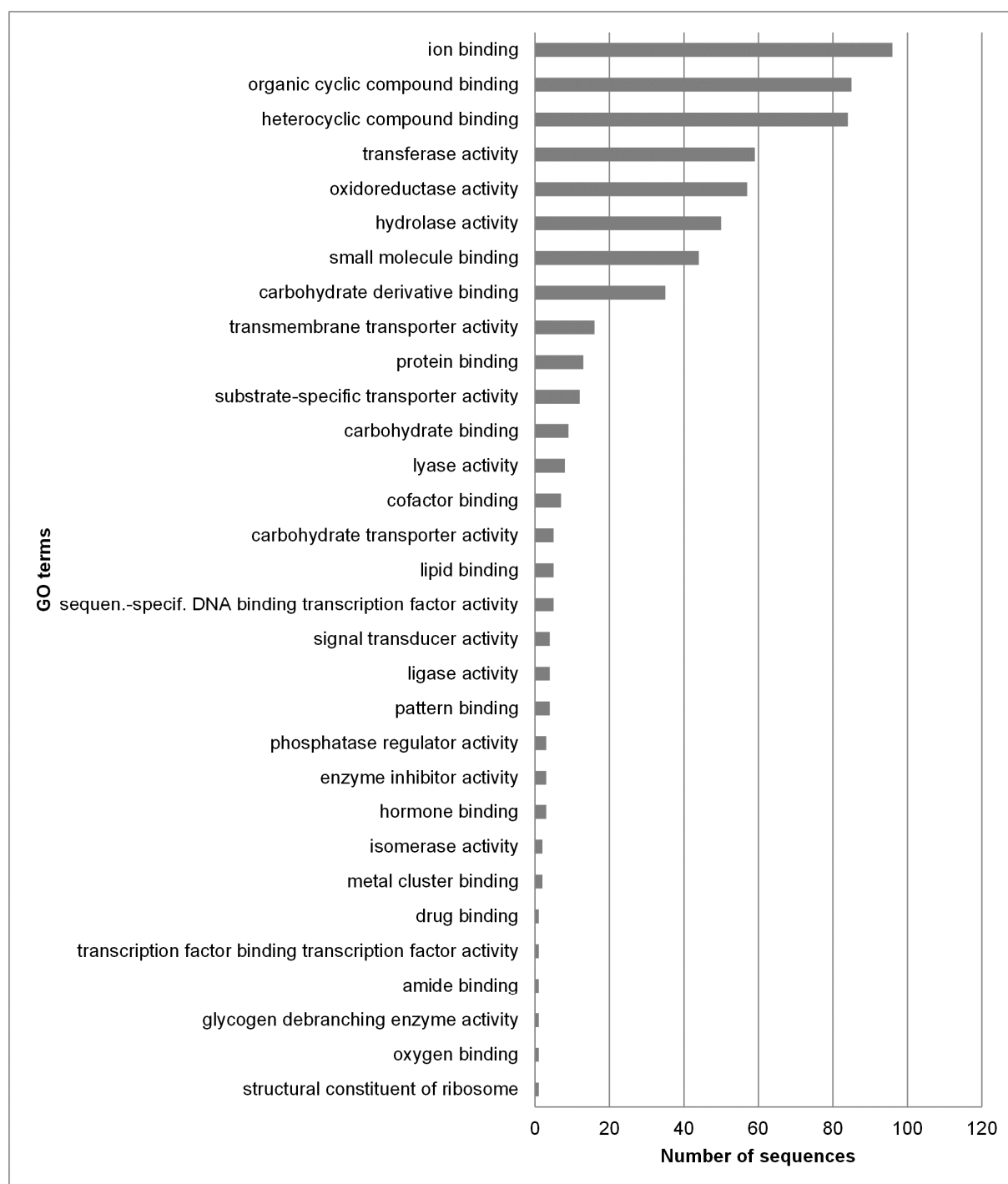

## Sampling day July 26

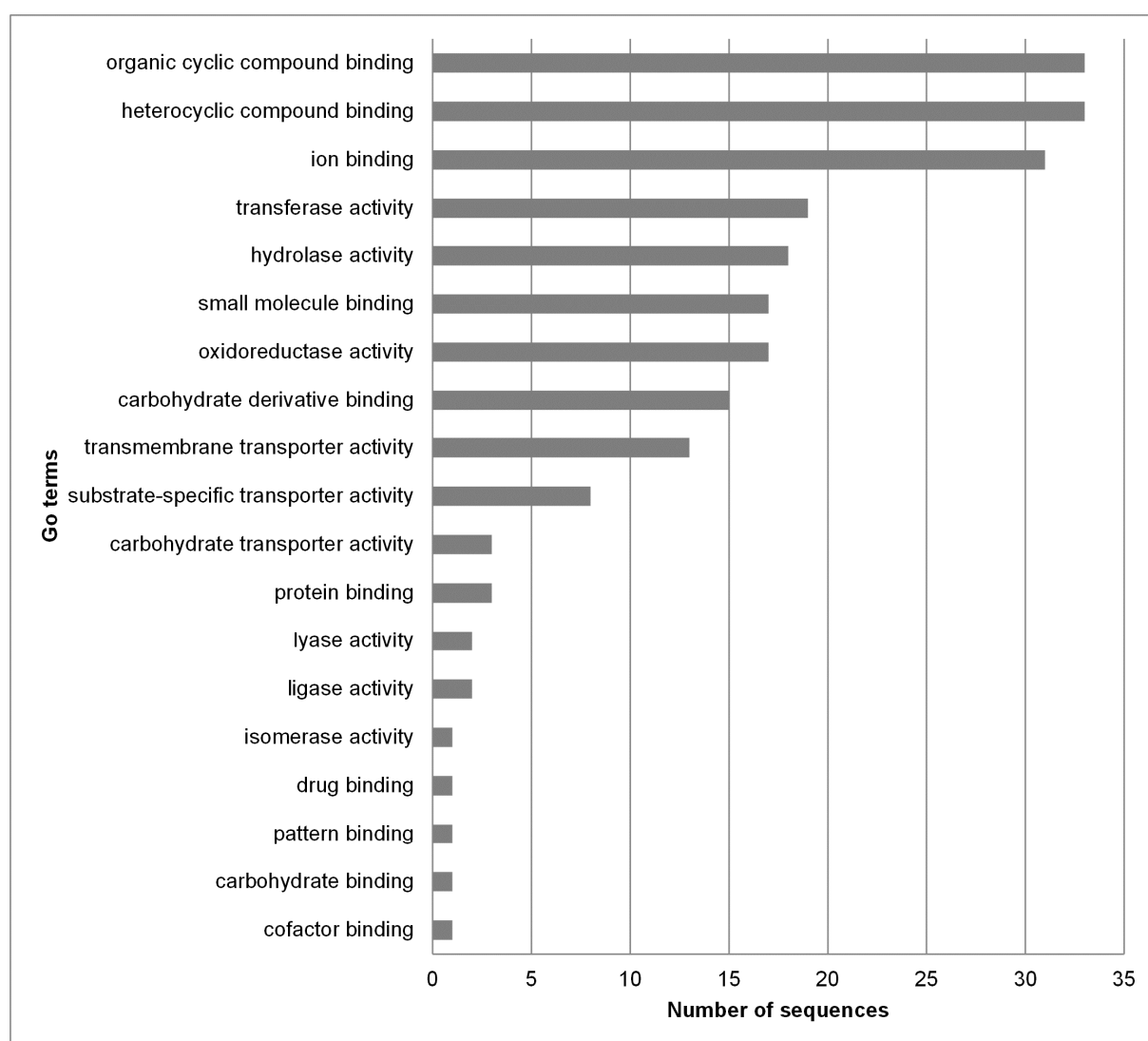

Supplement: S6 File — (PDF) [file pone.0184167.s006.pdf]
